# The topoisomerase 3 zinc finger domain cooperates with the RMI1 scaffold to promote stable association of the BTR complex to recombination intermediates in the *Caenorhabditis elegans* germline

**DOI:** 10.1093/nar/gkac408

**Published:** 2022-05-27

**Authors:** Maria Rosaria Dello Stritto, Nina Vojtassakova, Maria Velkova, Patricia Hamminger, Patricia Ulm, Verena Jantsch

**Affiliations:** Department of Chromosome Biology, Max Perutz Laboratories, University of Vienna, Vienna Biocenter, Austria; Department of Chromosome Biology, Max Perutz Laboratories, University of Vienna, Vienna Biocenter, Austria; Department of Chromosome Biology, Max Perutz Laboratories, University of Vienna, Vienna Biocenter, Austria; Department of Chromosome Biology, Max Perutz Laboratories, University of Vienna, Vienna Biocenter, Austria; Department of Chromosome Biology, Max Perutz Laboratories, University of Vienna, Vienna Biocenter, Austria; Department of Chromosome Biology, Max Perutz Laboratories, University of Vienna, Vienna Biocenter, Austria

## Abstract

Homologous recombination is the predominant DNA repair pathway used in the gonad. Of the excess DNA double-strand breaks formed in meiosis, only a subset matures into crossovers, with the remainder repaired as non-crossovers. The conserved BTR complex (comprising Bloom helicase, topoisomerase 3 and RMI1/2 scaffold proteins) acts at multiple steps during recombination to dismantle joint DNA molecules, thereby mediating the non-crossover outcome and chromosome integrity. Furthermore, the complex displays a role at the crossover site that is less well understood. Besides catalytic and TOPRIM domains, topoisomerase 3 enzymes contain a variable number of carboxy terminal zinc finger (ZnF) domains. Here, we studied the *Caenorhabditis elegans* mutant, in which the single ZnF domain is deleted. In contrast to the gene disruption allele, the *top-3-ZnF* mutant is viable, with no replication defects; the allele appears to be a hypomorph. The TOP-3-ZnF protein is recruited into foci but the mutant has increased numbers of crossovers along its chromosomes, with minor defects in repressing heterologous recombination, and a marked delay in the maturation/processing of recombination intermediates after loading of the RAD-51 recombinase. The ZnF domain cooperates with the RMI1 homolog RMH-2 to stabilize association of the BTR complex with recombination intermediates and to prevent recombination between heterologous DNA sequences.

## INTRODUCTION

Homologous recombination (HR) is a high-fidelity DNA repair pathway used to repair DNA damage, as well as programmed DNA lesions during meiosis. After double-strand break (DSB) induction, single-stranded 3′ overhangs are produced by resection. Once coated with the strand invasion protein RAD-51, the overhangs invade homologous DNA sequences to form strand invasion displacement D-loops. After extension by DNA synthesis, D-loops can either be pulled out of the heteroduplex, whereupon they re-anneal to form a non-crossover (NCO) via synthesis-dependent strand annealing (SDSA); or undergo second-end capture to generate joint DNA molecules that can mature into a crossover (CO), which mediates reciprocal exchange of chromatid portions. In meiosis, the number of induced DSBs exceeds the final number of COs, which guarantees the formation of at least one obligate CO per chromosome, and mechanisms exist to control the balance between CO and NCO formation. The recombination intermediates selected to differentiate into a CO are stabilized and protected by numerous pro-CO factors, such as the MutSy complex-like MSH-4/5 and the cyclin-related protein COSA-1/CNTD1 and the ubiquitin/sumo ligases ZHP-3/4 (1,2).

Together with cohesion, COs form the basis of the chromosomal tether between the homologs. In the absence of COs, unconnected parental homologous chromosomes (called univalents) segregate randomly during the first meiotic division. Joint DNA molecules are resolved by highly redundant endonucleases: depending on how these act upon the joint molecules, they generate either CO or NCO outcomes (3).

The BTR complex (comprising Bloom helicase, topoisomerase 3 and RMI1/RMI2 scaffold proteins) can also disassemble joint DNA molecules (for reviews see (4,5)). Based on *in vitro* reconstitution assays and structural studies, a model has been proposed in which the Bloom helicase unwinds the DNA in joint molecules by pushing the junctions of the joint molecules toward each other until a single hemicatenane remains; this is then removed by the topoisomerase together with the RMI1/RMI2 scaffold proteins through single-stranded DNA cleavage, DNA strand passage and nick re-sealing through tyrosine transesterification (6,7). In mitotic cells, this is the major pathway used to repair DNA lesions to generate NCOs, and patients with a mutation in any subunit of the BTR complex display elevated CO levels, chromosome instability and cancer predisposition (8,9). Monitoring of meiotic recombination intermediates in yeast has revealed that the BTR complex is important for D-loop reversion (10,11); this activity has also been demonstrated *in vitro* (12). Multi-joint molecules involving more than two chromatids can be prevented by BTR-mediated reversion of D-loops engaging with multiple chromatids. In yeast, the resolution of aberrant joint molecules requires a non-canonical resolvase such as Mus81 to form COs and NCOs or the joint action of Rmi1/topoisomerase 3. Persistent joint structures prevent chromosome segregation, leading to meiotic catastrophe. Resolvase-resistant structures include extended D-loops involving homologous and/or heterologous chromosomes or other branched DNA structures (10,11,13). Sgs1, the yeast Bloom helicase, is also involved in reversing strand invasion events into homeologous sequences during mitosis (14). Moreover, the germline activities of *C. elegans* BTR complex proteins suppress heterologous recombination to prevent chromosome translocations and genome rearrangements (15,16). Therefore, the BTR complex participates in multiple steps during meiotic recombination in many organisms. Functional analysis of BTR complex mutants has shown that the complex has separable roles in CO and NCO formation and influences the number and placement of the CO sites along the chromosomes (10,11,17–30).

Analysis of *C. elegans* mutants showed that the BTR complex proteins HIM-6 (Bloom helicase), RMH-1 (one of two RMI1 homologs) and RMIF-2 (the RMI2 homolog) provide both CO and NCO activities and prevent CO formation at chromosome centers (16,21,23,24,31), see Supplementary Figure S1A for a summary table for the *C. elegans* BTR complex protein nomenclature). The second RMI1 homolog, RMH-2, makes little contribution to meiosis by itself, as evidenced by the high hatch rates of embryos and lack of univalent chromosomes in the *rmh-2* mutant, in contrast to the *him-6*, *rmh-1* and *rmif-2* mutants. However, the *rmh-1; rmh-2* double mutants die as embryos, indicating a degree of functional overlap (21).

Topoisomerase 3 contains several highly conserved protein domains, including the topoisomerase domain with the TOPRIM and catalytic domains, and non-catalytic domains that include a variable number of C-terminal zinc fingers (ZnFs): the number ranges from zero in *Saccharomyces cerevisiae* and one in *C. elegans* to five in mammals (32) (See supplementary Figures S1B and S1C for the zinc finger domains in other organisms). Biochemical reconstitution assays using mammalian proteins have so far omitted the ZnF domain, indicating that it is not essential for decatenation and reversing D-loop formation *in vitro*. Suggested activities of the ZnF domain include DNA binding (33), a distinct activity in D-loop discouragement (25) and a recruitment activity required for the proper processing of Holliday junction intermediates (17).

Here, germline analysis of a *top-3* ZnF deletion mutant shows that it is a hypomorph, with minor defects in discouraging heterologous recombination events and elevated numbers of COs. We found that the ZnF domain is dispensable for TOP-3 activity in the mitotic progenitor cell compartment of the germline. Recombination foci, representing CO and NCO intermediates decorated by MSH-5 or RMH-1, were delayed and reduced in numbers. Nevertheless, formation of the six obligate COs was reflected in the high viability rate for the mutant. TOP-3 protein that is missing the ZnF domain was recruited to recombination foci and did not disrupt RMH-1 or HIM-6 localization during meiosis. We found that RMH-2 (the RMI1 homolog) and TOP-3 interact and suggest that they might form a subcomplex to perform specific activities. RMH-2 and the TOP-3 ZnF domain cooperate to localize the BTR complex and other recombination proteins, such as MSH-5, to the intermediates formed in early and mid-pachynema. We further observed that RMH-2 and the TOP-3 ZnF domain synergize to prevent heterologous recombination. We propose that the TOP-3 ZnF domain helps to stabilize the BTR complex at recombination sites.

## MATERIALS AND METHODS

### Experimental model and worm strains

All worm strains were maintained at 20°C (34) on Nematode Growth Medium (NGM) agar plates seeded with *Escherichia coli* OP50. Hermaphrodites were used in all experiments unless otherwise stated. The N2 Bristol strain was used as the wild-type (wt) control. For the strain list, see the Supplementary Materials and Methods.

### Cytology

Immunofluorescence analysis of worms at 20–24 h post-L4 stage was performed as previously described (35).

Gonads were dissected in 1× PBS on poly-l-lysine-coated slides, fixed with 1% paraformaldehyde in 1× PBS for 5 min at room temperature and then frozen in liquid nitrogen. After freeze-cracking, slides were incubated in methanol at −20°C for 1 min and then washed three times in PBS containing 0.1% Tween-20 (1× PBST) for 5 min at room temperature.

Non-specific binding sites were blocked by incubation in 1% BSA in 1× PBST for at least 30 min. Primary antibodies diluted in 1× PBST were applied and incubated overnight at 4°C. Slides were then washed three times in 1× PBST at room temperature and secondary antibodies were applied for 2 h. After three washes in PBST, 2 μg/ml DAPI was applied for 1 min and then slides were washed for at least 20 min in 1× PBST and mounted with Vectashield (36).

GFP::MSH-5 was detected as previously described for GFP::CKU-80 (36). Briefly, 10–15 young adult worms were dissected on poly-l-lysine-coated slides in 1× egg buffer containing 0.1% Tween-20 and immediately frozen in liquid nitrogen. After freeze-cracking, slides were incubated in methanol at −20°C for 1 min. Gonads were then fixed with 4% paraformaldehyde in 100 mM K_2_HPO_4_ pH 7.4 for 15 min, washed three times in 1× PBST and incubated with DAPI.

For high-resolution imaging, gonad spreads were stained as previously described (37). Gonads of 50 worms were dissected in 5 μl dissection solution (10% Dulbecco's modified Eagle's medium containing 0.1% Tween-20) on ethanol-washed 22 × 40 mm coverslips. Spreading solution (50 μl total volume: 32 μl fixative (4% paraformaldehyde and 3.2% sucrose in water) containing 16 μl Lipsol solution (1% lipsol in water) and 1% Sarcosyl solution (2 μl)) was added to dissected gonads, which were immediately spread across the whole coverslip using a pipette tip. Coverslips were dried overnight at room temperature, incubated in methanol at −20°C for 20 min and washed three times in 1× PBST. They were then incubated in 3% BSA in 1× PBS for at least 30 min before overnight incubation in primary antibody diluted in 1× PBST.

Primary antibodies were: rabbit anti-RAD-51 (1:1000 dilution; a gift from M. Zetka,), rabbit anti-HA (pre-absorbed to wt worms lysate, 1:100 dilution; Sigma-Aldrich, cat. #H6908); rabbit anti-OLLAS (pre-absorbed to wt worms, 1:1000 dilution, GenScript cat. #A01658); mouse anti-GFP (1:500 dilution, Roche cat. #11814460001), mouse anti-FLAG (pre-absorbed to wt worms; 1:200 dilution, Sigma cat. # F3165), guinea pig anti-HTP-3 (1:500 dilution; a gift from Yumi Kim); chicken anti-SYP-1 (1:500 dilution a gift from E. Martinez-Perez) and rabbit anti-mCherry (1:1000 dilution a gift from Alex Dammermann), anti-SUN-1pS8 (1:700 dilution (38)). Appropriate secondary antibodies conjugated to Alexa Fluor 488 or 594 were used at a dilution of 1:500 or those conjugated to Alexa Fluor 647 were used at a dilution of 1:250.

To quantify DAPI bodies at diakinesis, gonads were dissected from worms at 20–24 h post-L4 stage. For image acquisition and focus quantification, see the Supplementary Materials and Methods.

### Viability analysis

The analysis used single L4 hermaphrodites grown on individual NGM plates at 20°C. For details, see the Supplementary Materials and Methods.

### Gene editing using the CRISPR/Cas9 system

*top-3-ZnF, top-3::ha, rmh-2::flag* and *rmh-2(jf168)* strains were generated using a published protocol (39).

To generate the *top-3-ZnF* mutant, the amino acid 715 sequence 5′-TTC-3′ was modified to 5′-TAA-3′ to generate a stop codon. The repair template was also modified to remove a restriction site: the HpaII 5′-CCGG-3′ sequence was changed to 5′-CCAG-3′ for genotyping the strain. The repair template contains a 35-bp overlap with the *top-3* sequence.

The null *rmh-2* allele *jf168* was generated using two crRNAs: the first was located 22 bp before the 5′UTR and the second 29 bp before the 3′ stop codon. The repair template contains a 90 bp overlap with the *rmh-2* sequence.

The *top-3::ha* and *top-3-ZnF::ha* strains were generated using the repair template containing a 35 bp overlap to the *top-3* sequence. The HA sequence was inserted into an internal DNA sequence (between codons Gly 635 and Gly 636).

The *top-3-ZnF^4CtoA^::ha(jf219)* contains the following 4 amino acid transitions from cysteine to alanine: *C716A, C718A, C743A, C753A*. It was generated using a 200bp repair template in which all the cysteine residues of the Zinc finger domain of *top-3* (TGC/TGT) were mutated to alanine (GCG). The repair template contains around 30-bp overlap with the *top-3* sequence. Two crRNA were used to generate the mutations. The 5′ crRNA was the same used to generate the *top-3-ZnF* allele. The mutation of the cystein in position 718 aa generated a restriction site (KasI) that was used for genotyping of the strain.

The *rmh-2::flag* tagged line was generated by inserting a 3 × FLAG sequence (5′-GACTACAAAGACCATGACGGTGATTATAAAGATCATGATATCGATTACAAGGATGACGATGACAAG-3′) and linker (5′-GGTGGCAGTGGA-3′) prior to the stop codon.

The injection mix was as previously described (39) and included the following guide RNAs (crRNAs).

- *top-3-ZnF*: 5′-AAAAGAACAGGAAGAGGAAGAGG-3′
- *rmh-2(jf168)*: 5′crRNA, 5′-AATGTTGCGGTGTCTGTGCGCGG-3′; 3′crRNA, 5′-TTTTAATTGATCTTACGACGGGG-3′
- *top-3::ha/top-3-ZnF::ha*: 5′-CCTGGAGGTGGTGGTGGGGGAGG-3′
- *rmh-2::flag*: 5′-CTGTTACATGTTCGAGAATTAGG-3′.
- *top-3-ZnF^4CtoA^(jf219)*: 5′crRNA, 5′-AAAAGAACAGGAAGAGGAAGAGG-3′; 3′crRNA, 5′-AAAAATGTAATTTCTTTAAATGG-3′

To generate *rmh-2::mCherry*, two homology regions (left and right) in the *rmh-2* locus were amplified from genomic DNA. The right homology region was composed of two fragments (369 and 288 bp) fused via PCR; the same strategy was used for the left homology region (by combining two fragments of 317 and 355 bp). The homology regions were fused to the mCherry sequence and cloned into a pGEM-T Easy vector. The construct was sequence verified and injected into *unc-119(ed9)* III worms. The injection mix comprised guide RNA (20 ng/μl each), repair template (100 ng/μl), co-injections markers pCFJ90 and pGH8 (5 ng/μl each), and the gene of interest with the UNC-119 cloned into pGEM-T Easy vector (20 ng/μl).

The two guides used for injections were inserted into the pU6::klp-12 sgRNA vector (Addgene 46170), as previously described (40).

sgRNA I: 3′-CCACCAAATAAAGTGCTGGAATA-5′

sgRNA II: 5′-GACATCAGAAGCACAAAGGGTGG-3′.

CRISPR events were followed by PCR analysis and, for *top-3-ZnF*, by digestion with HpaII restriction enzyme or for *top-3-ZnF^4CtoA^(jf219)* with KasI restriction. All strains were sequence verified and outcrossed against the wt before analysis.

### Recombination frequency and CO analysis

The recombination frequency and CO number and position in wt, *top-3-ZnF* and *rmh-2(jf168)* were determined as previously described (16) using the *C. elegans* Hawaiian and Bristol hybrid strains. Specifically, we injected the mutation of interest into the Hawaiian strain and subsequently crossed it with the same mutation in the Bristol strain to generate the Hawaiian/Bristol hybrid. F1 progeny (in which the recombination events had taken place) were mated with Bristol males expressing a visible marker to monitor recombination events occurring exclusively during oogenesis. F2 generation worms heterozygous for visible marker expression, were lysed and differences between Bristol and Hawaiian were detected by PCR and subsequently SNP analysis of chromosomes V and IV. Oligonucleotide sequences and chromosome positions were published previously (16).

### Heterologous recombination assay

We used a published assay to examine the rate of heterologous recombination in different genetic backgrounds (wt, *top-3-ZnF, rmh-2(jf94), rmh-2(jf168), top-3-ZnF; rmh-2(jf168)*) (15). A *C. elegans* strain in which one copy of chromosome II is marked with the semi-dominant *dpy-25* mutation and the second contains the *mIn1* inversion (including a recessive *rol-1* mutation and a semi-dominant insertion of a GFP-expressing transgene) was used to score for recombinant progeny. If no recombination occurs within the *mIn1* inversion, a heterozygous *dpy-25/mIn1* [*rol-1*, GFP] II hermaphrodite would produce the following phenotypes in the progeny: 50% were heterozygous *dpy-25/mIn1* [*rol-1*, GFP] (phenotype: mild-dpy, non-rol, mild-GFP), 25% were homozygous *dpy-25/dpy-25* (phenotype: dpy, non-rol, non-GFP) and 25% were homozygous *mIn1* [*rol-1*, GFP]*/mIn1* [*rol-1*, GFP] (phenotype: non-dpy, rol, bright-GFP). If CO occurs between the *mIn1* [*rol-1*, GFP] sequence and chromosome II (containing the semi-dominant *dpy-25* mutation), different combinations of phenotypes would be produced in the progeny, such as dpy, non-rol and mild GFP; mild dpy, non rol and bright GFP, mild dpy, non rol and non GFP or non-dpy, rol and mild GFP. We crossed the mutant of interest with the strain containing *mln1* [*rol-1*, GFP] and scored the progeny for recombination events as detected by the phenotype.

### Yeast two hybrid assays

Yeast two hybrid assays were conducted using a published protocol (41). For details, see the Supplementary Materials and Methods.

### Statistical analysis

Statistical analyses included the Mann–Whitney test, chi-squared test and Fisher-exact test using Prism 6 software (GraphPad) and Microsoft Excel. Numbers were recorded and error bars (mean and standard deviation (SD)) and statistically significant differences were determined. Supplementary Table 1 indicates the specific *P* values for the indicated graphs.

### Biochemical studies

#### Nuclei isolation

For whole-cell protein extraction, 200 worms at the 24-h post-L4 stage were picked into 32 μl 1× Tris-EDTA buffer containing 1× Protease Inhibitor Cocktail (Roche cat. #11836170001) and freeze/thawed three times in liquid nitrogen. After thawing, 8 μl 5× Laemmli buffer was added and samples were boiled at 95°C for 10 min prior to the analysis by western blotting.

Fractionated proteins were generated as previously described (42). For this, one-sixth of a starved 60 mm plate was transferred onto a 100 mm plate and worms were grown at 20°C for 3 days. Worms were then collected in M9 buffer and washed at least three times (sedimented by gravity at room temperature) to remove most of the OP50 bacteria. The final worm pellet was frozen at −80°C in 3 ml NP buffer (10 mM HEPES–KOH pH 7.6, 1 mM EGTA, 10 mM KCl, 1.5 mM MgCl_2_, 0.25 mM sucrose, 1 mM PMSF) containing Protease Inhibitor Cocktail (Roche). Each fractionation was made from a 1 ml worm pellet obtained from 30–40 OP50-seeded 100 mm NGM plates.

To isolate the nuclei, worms were disrupted using a cooled metal Wheaton tissue grinder and the suspension was filtered using first a 100 μm mesh and then a 40 μm mesh. The filtered suspension was clarified at 300 *g* for 2 min at 4°C, and the supernatant from this step, containing the nuclei, was centrifuged at 2500 *g* for 10 min at 4°C. The supernatant was the cytosolic fraction, and the pellet contained the germline nuclei. The soluble and insoluble nuclear fractions were separated using a Qproteome Nuclear Protein Kit (Qiagen cat. #37582) according to the manufacturer's instructions.

#### Immunoprecipitation

A total of 650 μg extract from pooled soluble nuclear and insoluble fractions was used for each assay. HA-tagged proteins were immunoprecipitated with 20 μl HA magnetic beads (Pierce #88836) and mCherry-tagged proteins were immunoprecipitated with 25 μl RFP-trap_MA (Chromotek # rtma-10). For all immunoprecipitation experiments, beads pre-equilibrated in buffer D (20% glycerol, 0.2 mM EDTA pH 8, 150 mM KCl, 20 mM HEPES–KOH pH 7.9, 0.2% Triton X-100, supplemented with Protease Inhibitor Cocktail (Roche)) were incubated with worm extracts for 4 h at 4°C with mild agitation. The beads were then washed three times in buffer D for 7 min each. Bound proteins were eluted from HA-beads under acidic conditions: 30 μl glycine (100 mM, pH 2) was added to the beads and rotated for 10 min at room temperature. The beads were then separated magnetically and the supernatant (containing the target antigen) was transferred to a new tube. To neutralize the low pH, 4.5 μl 1 M Tris pH 9.2 was added to each sample. The eluates were analyzed by western blotting.

For the RFP-traps, beads were separated magnetically and bound protein was eluted by boiling the beads for 10 min at 99°C. The eluates were analyzed by western blotting.

#### Western blotting

Samples were boiled for 10 min and proteins were separated on precast 4–20% gradient polyacrylamide gels (Bio-Rad Laboratories, cat. #4561094) under denaturing conditions (1× SDS running buffer: 0.2501 M of Tris, 1.924 M of glycine and 0.0347 M of SDS). Proteins were transferred onto PVDF membrane (Amersham Hybond cat. #10600023) for 1 h at 4°C at 100 V in 1 × Tris-glycine buffer (125 mM Tris and 960 mM Glycine) containing 20% methanol. After washing three times in 1× TBS containing 0.1% Tween-20 (1 × TBST) for 5 min at room temperature, membranes were blocked for at least 30 min in 5% milk in 1× TBST. Primary antibodies were diluted in 5% milk in 1× TBST and incubated with membranes overnight at 4°C. Membranes were then washed in 1× TBST three times for 10 min each and then incubated with the appropriate HRP-conjugated secondary antibody diluted in 5% milk in TBST 1× for 2 h at room temperature. After three further washes, membranes were incubated for 1 min at room temperature with ECL Star Enhanced Chemiluminescent Substrate (Euroclone cat #EMP001005) or, for low abundance proteins, for 2 min with WesternBright Sirius HRP substrate (Advansta K-12043-D20) and proteins were visualized with a ChemiDoc system (Bio-Rad Laboratories).

Primary antibodies were: mouse anti-HA (1:1000 dilution; Cell Signaling, cat. #2367); mouse anti-FLAG (1:1000 dilution; Sigma, cat. #F3165), rabbit anti-mCherry (1:7000 dilution; a gift from Alex Dammermann); goat anti-actin (1:3000 dilution; Santa Cruz Biotechnology, cat. #sc-1616) and rabbit anti-histone 3 (1:100 000 dilution; Abcam, cat. #ab1791). Secondary antibodies were anti-rabbit HRP (1:25 000 dilution; Pierce, cat. #31460), anti-mouse HRP (1:2500 dilution; Cell Signaling, cat. #7076) and anti-goat HRP (1:5000 dilution; Santa Cruz Biotechnology, cat. #sc-2020).

## RESULTS

### The *C. elegans* TOP-3 ZnF domain is not required for viability and does not affect protein stability

**Figure 1.**
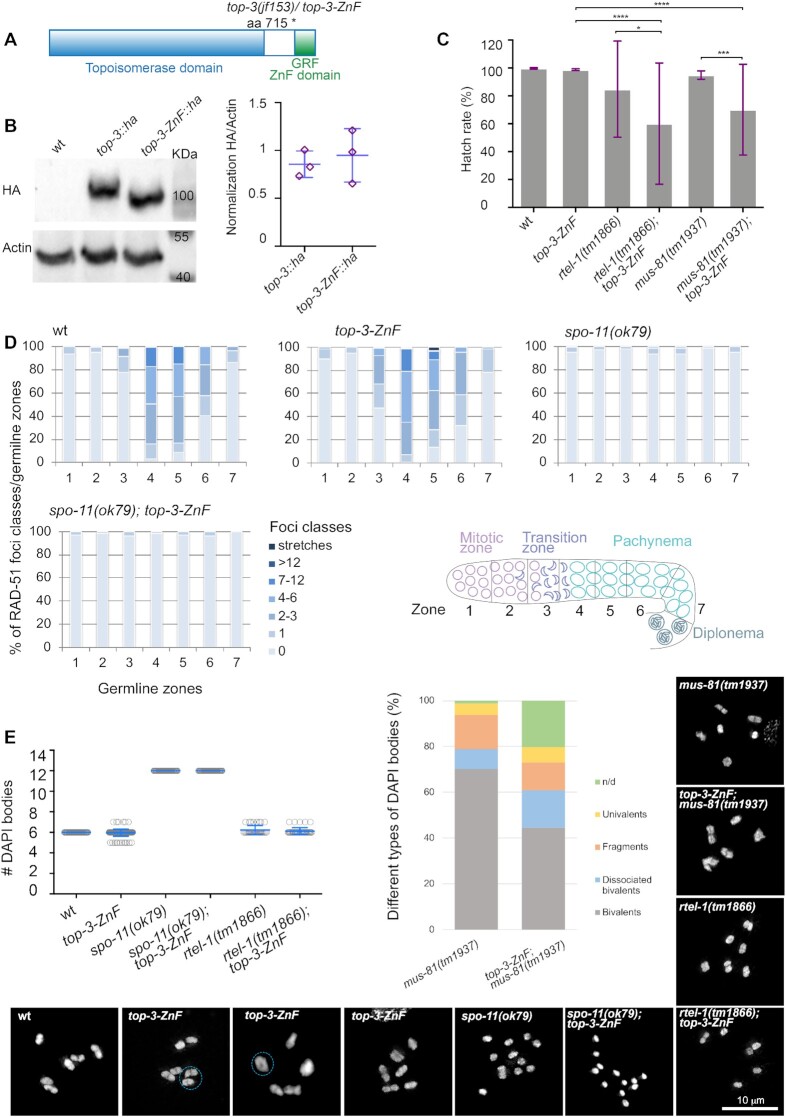
*t* *op-3-ZnF* is a hypomorph allele. (**A**) Schematic representation of the *top-3* gene, depicting the conserved topoisomerase domain (light blue) and the GRF zinc finger (ZnF) motif (green). The *jf153* (*top-3-ZnF*) allele encodes a truncated protein that lacks the ZnF motif. (**B**) Western blot analysis of TOP-3::HA and TOP-3-ZnF::HA in whole worm extracts. wild-type (wt) worms were used to control for the specificity of the antibody. The TOP-3::HA protein is 100 KDa in size and the TOP-3-ZnF::HA protein lacks 45 amino acids. Actin was used as the loading control. Right panel: western blot quantification of three biological replicates per sample. Error bars indicate the mean and SD. (**C**) Hatch rates for the indicated genotypes: wt, 99.8% ± 0.3%, *n* = 18; *top-3-ZnF*, 98.8% ± 0.7%, *n* = 17; *rtel-1(tm1866)*, 84.7% ± 34.3%, *n* = 8; *rtel-1(tm1866); top-3-ZnF*, 60.1% ± 43.4%, *n* = 9; *mus-81(tm1937)*, 95.0% ± 3.0%, *n* = 8; *mus-81(tm1937)*; *top-3-ZnF*, 70.1% ± 32.5%, *n* = 7. *n* = number of hermaphrodites. The histogram indicates the mean ± (SD). * *P* = 0.02, *** *P* = 0.0003, **** *P* < 0.0001, as determined using the Mann–Whitney test; non-significant differences are not shown. (**D**) The percentage of RAD-51 foci classes in each of the seven zones of the gonad in the indicated genotypes. For each zone, the average number of RAD-51 foci/nucleus was calculated from three gonads per genotype. Bottom right panel: schematic representation of the *C. elegans* gonad divided into seven equal zones. (**E**) Quantification of DAPI bodies in the −1 diakinesis oocyte in the indicated genotypes. Mean ± SD and number (*n*) of DAPI bodies per genotype: wt, 6 ± 0, *n* = 50; *top-3-ZnF*, 5.96 ± 0.3, *n* = 154; *spo-11(ok79)*, 12 ± 0, *n* = 50; *spo-11(ok79); top-3-ZnF*, 12 ± 0, *n* = 50; *rtel-1(tm1866)*, 6.2 ± 0.4, *n* = 34; and *rtel-1(tm1866); top-3-ZnF*, 6.1 ± 0.3, *n* = 41. Right panel: qualitative analysis of the −1 diakinesis oocyte of the indicated genotypes. *n* = number of DAPI bodies and the percentage of the different types of DAPI bodies observed per genotype: *mus-81(tm1937)*, *n* = 90, bivalents = 70.2%, dissociated bivalents = 8.5%, DNA fragments = 14.9%, univalents = 5.3%, n/d (not defined) = 1.1%; *mus-81(tm1937); top-3-ZnF*, *n* = 50, bivalents = 44.6%, dissociated bivalents = 16.2%, fragments = 12.2%, univalents = 6.8%, n/d (not defined) = 20.3%. Insets show representative images of DAPI-stained diakinesis nuclei of the indicated genotypes. For *top-3-ZnF*, the dashed light blue circles indicate the presence of bridges and a doughnut-shaped bivalent.

The C-terminal domain of type 1A topoisomerases is less well conserved than the catalytic domain. In numerous organisms, the C-terminus contains multiple ZnF domains, at least three: the one directly after the topoisomerase catalytic domain is a four-cysteine zinc motif (Zn-C4) that is followed by two GRF motifs, defined by a central tandem glycine–arginine–phenylalanine conserved region (43). *C. elegans* TOP-3 contains one GRF-type ZnF domain (Supplementary Figures S1B and C). To investigate its function, we deleted the conserved ZnF domain of TOP-3 by introducing a premature stop codon (amino acid 715 to STOP; Figure 1A). We will refer to the new *top-3* allele, *jf153*, as *top-3-ZnF*. We also inserted an internal HA tag into both the wt and *top-3-ZnF* alleles, as previously described (16). Western blot analysis showed that the truncated protein is expressed at similar levels to the wt (Figure 1B).

The *top-3-ZnF* mutant displayed a hatching rate of 98.8% (±0.7 SD), which is similar to the wt (99.8% (± 0.5 SD); Figure 1C)); in striking contrast, the *top-3(jf101)* disruption allele has 100% embryonic lethality (36). Adult *top-3-ZnF* mutant worms laid an average of 245 (±33.7 SD) eggs, compared with 229 (±28.1 SD) in the wt. *top-3-ZnF* worms also displayed slightly elevated X chromosome non-disjunction, as revealed by the increased incidence of males (X/O genotype: 0.3% (±0.4 SD) versus 0.02% (±0.1 SD) in the wt). Moreover, the mutant had a larval arrest rate of 0.7% (±0.5 SD), whereas this phenotype is never seen in the wt. Taken together, these data indicate that *top-3-ZnF* mutants display only very mild meiotic and developmental defects.

### *top-3-ZnF* mutants display mild defects in the germline

To examine the kinetics of DSB repair events in *top-3-ZnF* worms, we analyzed RAD-51 focus dynamics during prophase I (44,45). Similar to the wt, in *top-3-ZnF* mutants RAD-51 foci appeared in the transition zone, peaked in early–mid pachynema and disappeared in late pachynema, likely due to the completion of repair (Figure 1D). No RAD-51 foci were observed in the absence of *spo-11*-dependent meiotic DSBs in the *top-3-ZnF; spo-11(ok79)* double mutant (Figure 1D and Supplementary Figure S1D). This indicates that in *top-3-ZnF* worms all premeiotic recombination intermediates are properly repaired before meiotic entry; therefore, the ZnF domain of TOP-3 is not required for premeiotic replication. In contrast, in the *top-3(jf101)* gene disruption allele RAD-51 marked recombination intermediates were observed throughout the germline, indicating the presence of unrepaired lesions in both mitotic and meiotic cells (36).

The readily accessible diakinesis chromosomes of *C. elegans* oocytes are a readout for the success of prophase I events (including recombination). We quantified the number of DAPI-stained bodies at diakinesis, equivalent to the six connected parental homologous chromosomes (bivalents) in the wt. In *top-3-ZnF*, we saw an average of 5.96 (±0.3 SD) DAPI-stained bodies at diakinesis, of which 6% were chromosomal bridges and 31% were ‘donut-shaped’ bivalents (i.e. bivalents with a visible hole in the middle), which are never observed in the wt (Figure 1E). Those could be chromosome pairs with more than one CO (see below for evidence of double COs in the mutant). The *top-3-ZnF; spo-11* double mutant had 12 DAPI-stained bodies, corresponding to the 12 achiasmatic chromosomes also seen in the single *spo-11* mutant (Figure 1E).

Previous studies have shown that in mutants with recombination defects the zone positive for the phosphorylation of SUN-1 Serine 8 is extended, indicating a delayed meiotic progression. Staining for SUN-1 pS8 revealed that the dynamics of phosphorylation and dephosphorylation of SUN-1 are identical in the mutant and wt (Supplementary Figure S2A).

*top-3-ZnF* worms differed phenotypically from those with the *top-3(jf101)* disruption allele, but also from the *rmh-1, rmif-2* and *him-6* mutants (16,21,24,31,36). *top-3-ZnF* mutants were viable, did not display severe chromosome mis-segregation (since they lacked joint structures and univalents at diakinesis, otherwhise observed in *top-3(jf101)*, *him-6* and *rmh-1* mutants, Supplementary Figure S2B) and had a low frequency (0.3%) of males, which is significantly lower than in mutants of other BTR complex proteins (*him-6* 6%, *rmh-1* 14%, *rmif-2* 10%; (16)). This suggests that the ZnF domain is dispensable for some activities of the BTR complex. In fact, *top-3-ZnF* mutants did not display interlocks (likely to derive from accumulated joint molecules), which were seen in the disruption allele *top-3(jf101)* upon high-resolution imaging (Supplementary Figure S2C). Therefore, this allele is amenable to a wide range of phenotypic analysis otherwise impossible to perform in the *top-3(jf101)* disruption allele due to the severity of its meiotic and mitotic phenotypes.

### Impairment of different recombination pathways highlights the hypomorphic phenotype of *top-3-ZnF*

Redundant pathways are responsible for D-loop discouragement in *C. elegans*, as in many other experimental systems. NCOs can stem from SDSA promoted by the D-loop reverting helicase RTEL-1 or from the dissolution of double Holliday junctions; both D-loop reversion and dissolution can be mediated by the BTR complex. The simultaneous impairment of these two repair pathways is lethal in worms: *rmh-1; rtel-1* double mutants are completely sterile (21) and only 7% of *him-6; rtel-1* offspring are viable (46). To understand whether the ZnF domain contributes to D-loop discouragement, we constructed the *top-3-ZnF; rtel-1* double mutant. Surprisingly, this mutant had a hatch rate of 60.1% (±43.2 SD), indicating that it is fertile but significantly less so than *rtel-1* single mutants (84.7% (±34.4 SD); Figure 1C). The average number of eggs laid per worm was similar between the single and double mutants (76.75 (±53.7 SD) for *rtel-1* and 79.4 (±54.2 SD) for *top-3-ZnF; rtel-1*), as was the number of larval arrests (14.2 (±6 SD) for *rtel-1* and 21.3 (±23.6 SD) for *top-3-ZnF; rtel-1*). Diakinesis chromosome structures were also similar between the single and double mutants (6.2 (±0.4 SD) for *rtel-1* and 6.1 (±0.3 SD) for *top-3-ZnF; rtel-1*; Figure 1E). These results indicate that the activities of the BTR complex are only partly blocked by removal of the TOP-3 ZnF domain.

When BTR complex activity is compromised, the MUS81 resolvase can process joint DNA intermediates, including blocked replication forks. In *Arabidopsis*, the T1 TOP3 ZnF domain is required for replication-associated DNA damage repair in parallel to the MUS81 pathway (17). We constructed the *mus-81; top-3-ZnF* double mutant to address whether the ZnF domain is also required for this activity in *C. elegans*. In *C. elegans*, the BTR complex might also work in parallel to MUS-81 to resolve joint molecules since the simultaneous impairment of *mus-81* and *top-3(jf101)* (a disruption allele) led to an accumulation of joint structures at diakinesis (36). The *mus-81; top-3-ZnF* double mutant had a hatch rate of 70.1% (±32.6 SD), which is significantly lower than those of *mus-81* (95.5% (±3 SD) and *top-3-ZnF* 98.8% (±0.7 SD). Cytological inspection of *mus-81; top-3-ZnF* diakinesis chromosomes revealed an aberrant morphology compared with *mus-81*, with higher rates of undefined chromatin masses (20.3% versus 1.1%), dissociated bivalents possibly arising from a failure in the resolution of joint molecules (16.2% versus 8.5%) and univalents (6.8% versus 5.3%) but less DNA fragmentation (12.2% versus 14.9%; Figure 1E). These findings suggest that recombination intermediates are less efficiently repaired in the double mutant. Taken together, the data indicate that the *top-3-ZnF* mutant behaves as a hypomorphic allele of *top-3*.

### *top-3-ZnF* mutants have more COs and a mild increase in heterologous recombination rate

**Figure 2.**
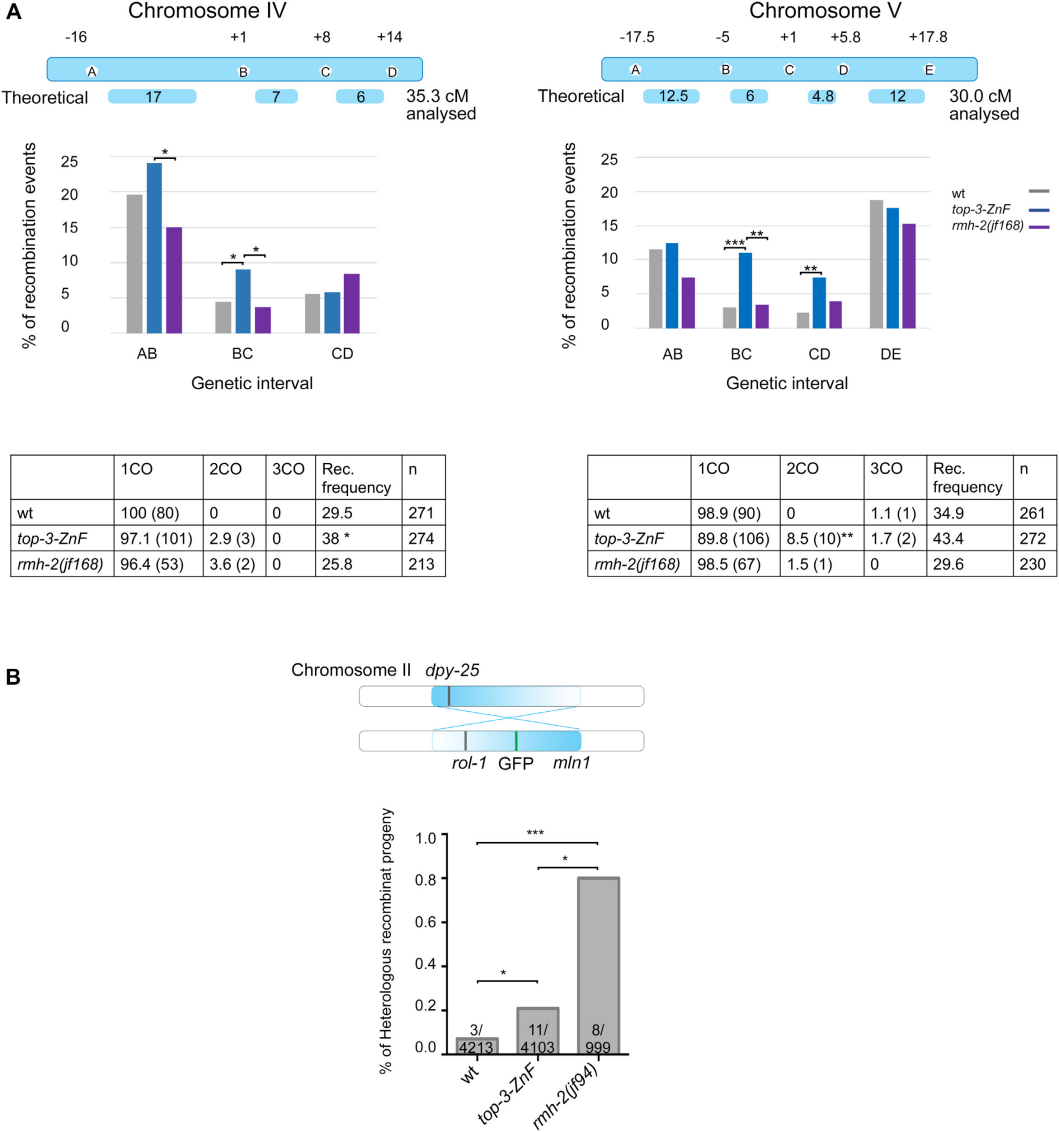
The*top-3-ZnF* mutant displays extra COs and low levels of heterologous recombination. (**A**) Top: schemes of chromosomes IV and V and the corresponding SNP locations used in the PCR-based recombination assay for wt, *top-3-ZnF* and *rmh-2(jf168)* worms. The scheme indicates the theoretical genetic distance (in cM) and the experimentally determined distance for each chromosome. The graph represents the percentage of recombination events in each genetic interval for Chromosome IV (left) and V (right). The statistical significance of differences between *top-3-ZnF* and wt (Chromosome IV, BC interval * *P* = 0.04; and Chromosome V, BC interval *** *P* = 0.0009, CD interval ** *P* = 0.0099) and between *rmh-2(jf168)* and *top-3-ZnF* (Chromosome IV, AB interval * *P* = 0.042, BC interval * *P* = 0.028; and Chromosome V, BC interval ** *P* = 0.003) was calculated using the χ^2^ test. Bottom: tables show the percentage of single (1CO), double (2CO) and triple (3CO) crossover, with the numbers in parenthesis, the recombination (Rec.) frequency as a percentage and the number of worms analyzed per genotype. The statistical significance of differences in recombination frequency between strains were calculated using Fisher's exact test (Chromosome IV: *top-3-ZnF* vs wt * *P* = 0.046, *top-3-ZnF* vs *rmh-2(jf168)* ** *P* = 0.0048; wt vs *rmh-2(jf168)* not significant (ns); Chromosome V: *top-3-ZnF* vs wt ns; *top-3-ZnF* vs *rmh-2(jf168)* ** *P* = 0.0016; wt vs *rmh-2(jf168)* ns). In Chromosome V, the percentage of crossovers that were double crossovers was significantly increased in *top-3-ZnF* compared with wt (** *P* = 0.0063), as calculated using the χ^2^ test. (**B**) Top: schematic representation of Chromosome II, showing the *mln1* inversion and phenotypic markers used for the recombination assay (recessive *rol-1* and semi-dominant GFP and *dpy-25*). Bottom: Percentage of recombinant progeny and the number of recombination events/total number of worms tested are shown for each genotype. Statistical analysis was done using Fisher's exact test *(top-3-ZnF* compared with wt * *P* = 0.033; *top-3-ZnF* compared with *rmh-2(jf94)* * *P* = 0.028; *rmh-2(jf94)* compared with wt *** *P* = 0.0002).

To investigate whether the TOP-3 ZnF domain regulates recombination, we compared recombination frequency and the chromosomal position of COs in mutant and wt worms by exploiting the different SNP profiles in the *C. elegans* ecotypes from Bristol (N2) (the more common laboratory strain) and Hawaii (CB4856) (47). We inserted a homozygous *top-3-ZnF* mutation into the Hawaiian strain and generated the Hawaiian/Bristol hybrid. In the progeny, we measured the frequency of recombination by analyzing four SNPs (i.e. three intervals) covering 35.3 cM of chromosome IV and five SNPs (i.e. four intervals) covering 30 cM of chromosome V (Figure 2A). The *top-3-ZnF* mutant had a significantly increased recombination frequency and an overall increase in the number of COs. The COs were aberrantly located also to chromosome centers (where they are usually excluded in the wt). The recombination landscape in this mutant is different from the one observed in *rmh-1* and *him-6*, where the COs were increased in the center region of the chromosome but were strongly reduced in the arm regions without any double COs appearance (21,48). In the *top-3-ZnF* mutant we observed an increase in double and triple COs compared with the wt: double COs comprised 2.9% of all COs on chromosome IV and 8.5% of all COs on chromosome V (with no double COs in the wt); and triple COs were only found on chromosome V, where they comprised 1.7% of all COs in the mutant and 1.1% of all COs in the wt (in wt may be due to a technical error) (Figure 2A and Supplementary Figure S3A). These results indicate that in *C. elegans* deletion of the TOP-3 ZnF domain impacts the number and distribution of COs, as previously reported for *Arabidopsis* (25,30).

During HR, strand invasion into heterologous sequences (i.e. regions with low sequence identity) is usually reversed to prevent genome rearrangements. In worms, this can be achieved by RTEL-1 and BTR complex proteins (15,16). Therefore, we analyzed heterologous recombination events in *top-3-ZnF* to determine whether the ZnF domain is also involved in rejecting erroneous, unproductive strand invasion. For this, we used the *mln1* inversion on chromosome II and assessed recombination events between heterologous regions by scoring the exchange of genetic markers: *dpy-25* on one side of chromosome II and recessive *rol-1* and semi-dominant GFP (flanking the *mln1* inversion) on the other side of chromosome II (Figure 2B and Supplementary Figure S3B) (15). Whereas in the wt these events were extremely rare (recombination rate, 0.07%; 4 out of 4213), in the *top-3-ZnF* mutant we found a slight but significant increase in the heterologous recombination rate (0.21%; 11 out of 4103). The rate of heterologous recombination events in the mutant differed from those in other BTR complex mutants, which had reproducible rates of approximately 7% (*rmh-1* and *him-6*) (15,16). This result suggests that 1) the ZnF domain in TOP-3 is mostly dispensable for D-loop reversions or 2) there is functional redundancy regarding D-loop reversion between the ZnF domain and another domain in TOP-3 or another BTR complex protein.

### The ZnF domain is essential for efficient recruitment of TOP-3 to early recombination intermediates

**Figure 3.**
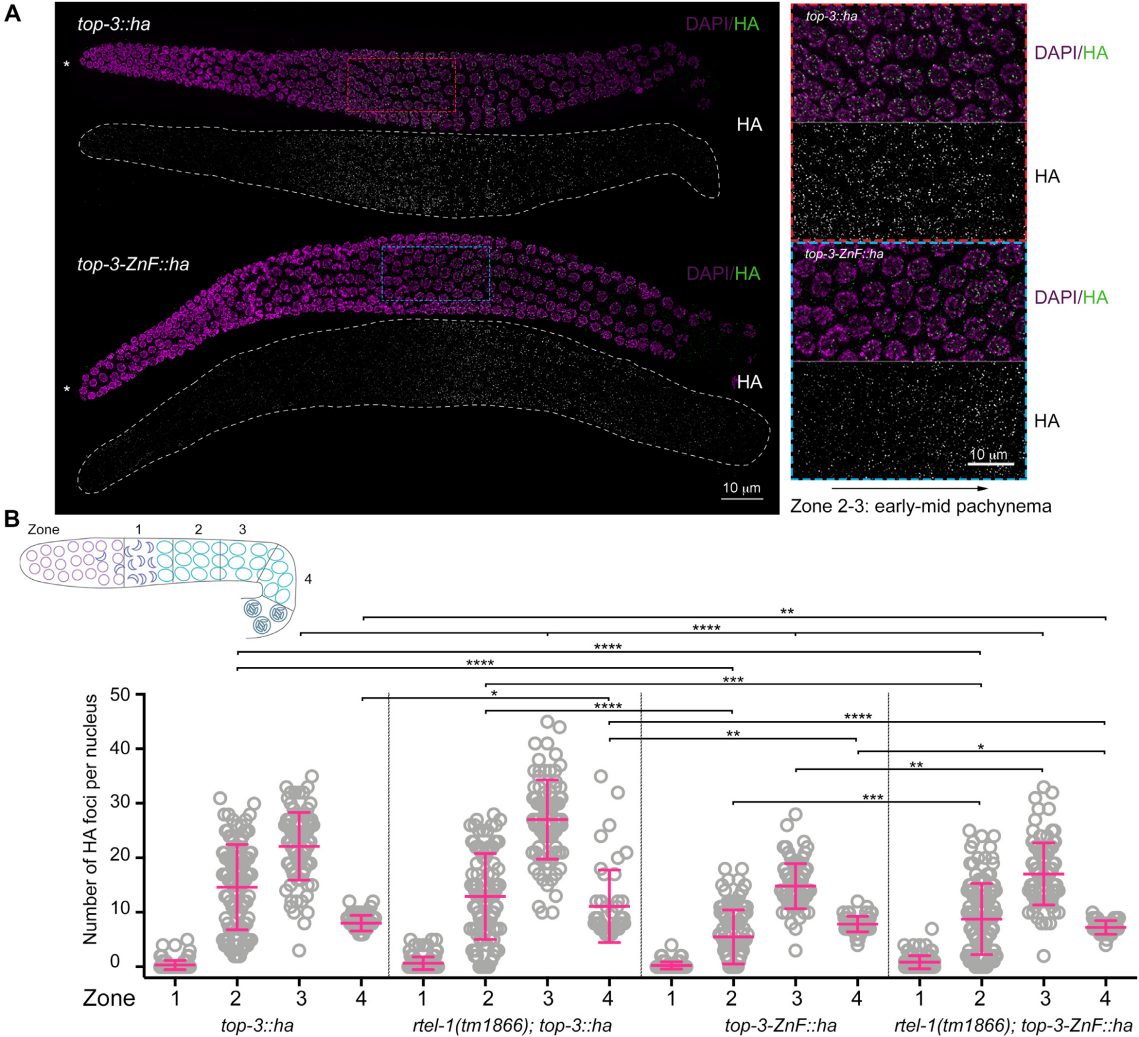
*top-3-ZnF* localizes to foci. (**A**) Representative images of *top-3::ha* (top) and *top-3-ZnF::ha* (bottom) gonads stained with HA (green) and DAPI (magenta). * Indicates the distal tip of the progenitor zone. Red and blue squares (dashed lines) indicate the enlarged regions on the right. (**B**) Schematic representation of the *C. elegans* gonad divided into four equal zones from the transition zone to late pachynema. Scatter plots show the number of HA foci per nucleus in the meiotic region of the gonad: zone 1 = transition zone; zone 2 = early pachynema; zone 3 = mid pachynema; and zone 4 = late pachynema. For each genotype, the mean ± SD number of foci in each zone are indicated, with *n* = the number of nuclei assessed. *top-3::ha*: zone 1, 0.3 ± 0.8, *n* = 141; zone 2, 14.7 ± 7.8, *n* = 110; zone 3, 22.1 ± 6.2, *n* = 89; and zone 4, 8 ± 1.4, *n* = 71. *rtel-1(tm1866); top-3::ha* zone 1, 0.7 ± 1.2, *n* = 141; zone 2, 12.9 ± 7.9, *n* = 98; zone 3, 27.1 ± 7.3, *n* = 88; and zone 4, 11.1 ± 6.6, *n* = 49. *top-3-ZnF::ha*: zone 1, 0.3 ± 0.6, *n* = 121; zone 2, 5.5 ± 5, *n* = 92; zone 3, 14.8 ± 4.1, *n* = 83; and zone 4, 7.8 ± 1.4, *n* = 63. *rtel-1(tm1866); top-3-ZnF::ha*: zone 1, 0.9 ± 1.2, *n* = 132; zone 2, 8.8 ± 6.5, *n* = 117; zone 3, 17.1 ± 5.7, *n* = 94; and zone 4, 7.2 ± 1.2, *n* = 45. Error bars indicate the mean and SD. Statistical significance was determined using the Mann–Whitney test (see Supplementary table 1 for more details of the statistical analysis).

The ZnF domain is assumed to bind to DNA (33), thereby targeting the topoisomerase to a specific recombination substrate (17). To gain insight into how deletion of the ZnF domain affects TOP-3 localization in the germline, we measured focus formation in a TOP-3::HA-tagged line (functionality of the strain is shown in Supplementary Figure S4A and B). For focus quantification, we divided the germline into four equal regions from the transition zone to late pachynema, with zone 1 corresponding to the transition zone, zone 2 to early pachynema, zone 3 to mid pachynema and zone 4 to late pachynema (the same approach was used to quantify recombination foci throughout the study). TOP-3 foci started to appear in early pachynema (zone 2), with an average of 14.7 (±7.8 SD) foci per nucleus; peaked in mid pachynema (zone 3), with 22.1 (±6.2 SD) foci per nucleus; and then declined by late pachynema (zone 4), with 8 (±1.4 SD) foci per nucleus (Figure 3A and B). TOP-3 was able to concentrate into foci even in absence of the ZnF domain (*top-3-ZnF::ha*), although with different dynamics compared with wt *top-3::ha*. In *top-3-ZnF::ha*, early TOP-3-ZnF foci were delayed and reduced in numbers compared with *top-3::ha*: in zone 2 (early pachynema), TOP-3-ZnF foci localized with an average of 5.5 (±5 SD) foci per nucleus, which was significantly lower than in *top-3::ha*; and similarly, in zone 3 (mid pachynema) the average of TOP-3-ZnF foci per nucleus was only 14.8 (±4.1 SD; Figure 3A and B). There was no significant difference in the numbers of late foci between *top-3::ha* and *top-3-ZnF::ha* (7.9 (±1.4 SD) foci per nucleus). We furthermore examined whether the region with the late foci would appear at the same time in the wt and the mutant. We did not see a difference between *top-3::ha* (32.9 (±1.7 SD)) and *top-3-ZnF::ha* (33.7 (±1.5 SD)), see Supplementary Figures S4C and S4D, where we measured the percentage of cell rows with late foci. We also generated a *top-3* allele, where the Zn finger domain was impaired by mutating the Zn coordinating cysteines to alanines, *top-3-ZnF^4CtoA^::ha* (see materials methods for details and Supplementary Figure S1B). HA immunostaining showed that in this allele foci appeared with a comparable delay as seen in the *top-3-ZnF* allele (Supplementary Figure S4E). The average region positive for HA in *top-3::ha* was 67.9% (±3 SD) while in *top-3-ZnF::ha* it amounted to 54.1% (±2 SD) and in *top-3-ZnF^4CtoA^::ha* to 56.8% (±2.5 SD), confirming a similar defect in the two alleles (Supplementary Figure S4F).

Therefore, although the ZnF domain is not strictly needed to concentrate TOP-3 into foci, it might have more prominent roles in recognition and/or stabilization of the BTR complex on early recombination intermediates or in processing these intermediates (from now on, we refer to early recombination intermediates as those foci present in early–mid pachynema).

Depletion of the RTEL-1 helicase increases the number of CO-precursor intermediates, which are marked by the BTR complex proteins (21). We also consistently observed an increased number of TOP-3 foci in mid–late pachynema in *rtel-1; top-3::ha*. In zone 2, the average number of foci per nucleus was similar between *rtel-1; top-3::ha* and *top-3::ha* (12.9 ± (7.9 SD) and 14.7 (± 7.8 SD) respectively); however, in zone 3, the peak number of foci per nucleus increased to 27.1 (±7.3 SD) in *rtel-1; top-3::ha*. A similar increase was also observed in zone 4: *top-3::ha* had 8 (± 4 SD) foci per nucleus, whereas *rtel-1; top-3::ha* had 11.1 (±6.6 SD) foci per nucleus (Figure 3B).

We generated the *rtel-1*; *top-3-ZnF::ha* mutant and observed that, although as expected, the number of foci was higher compared to *top-3-ZnF::ha*, the deletion of the ZnF domain delayed and reduced the number of early foci compared with the *rtel-1* single mutant: in *rtel-1; top-3-ZnF::ha*, zone 2 contained 8.8 (±6.5 SD) foci per nucleus, zone 3 contained 17.1 (±5.7 SD) foci per nucleus and zone 4 contained 7.2 (±1.2 SD) foci per nucleus. This observation supports the hypothesis that the ZnF domain of TOP-3 is required for accumulation of the complex onto the early recombination intermediates or for their timely processing, but is dispensable for its association with late recombination intermediates.

### The TOP-3 ZnF domain is partially required to target the BTR complex to a subset of recombination foci

**Figure 4.**
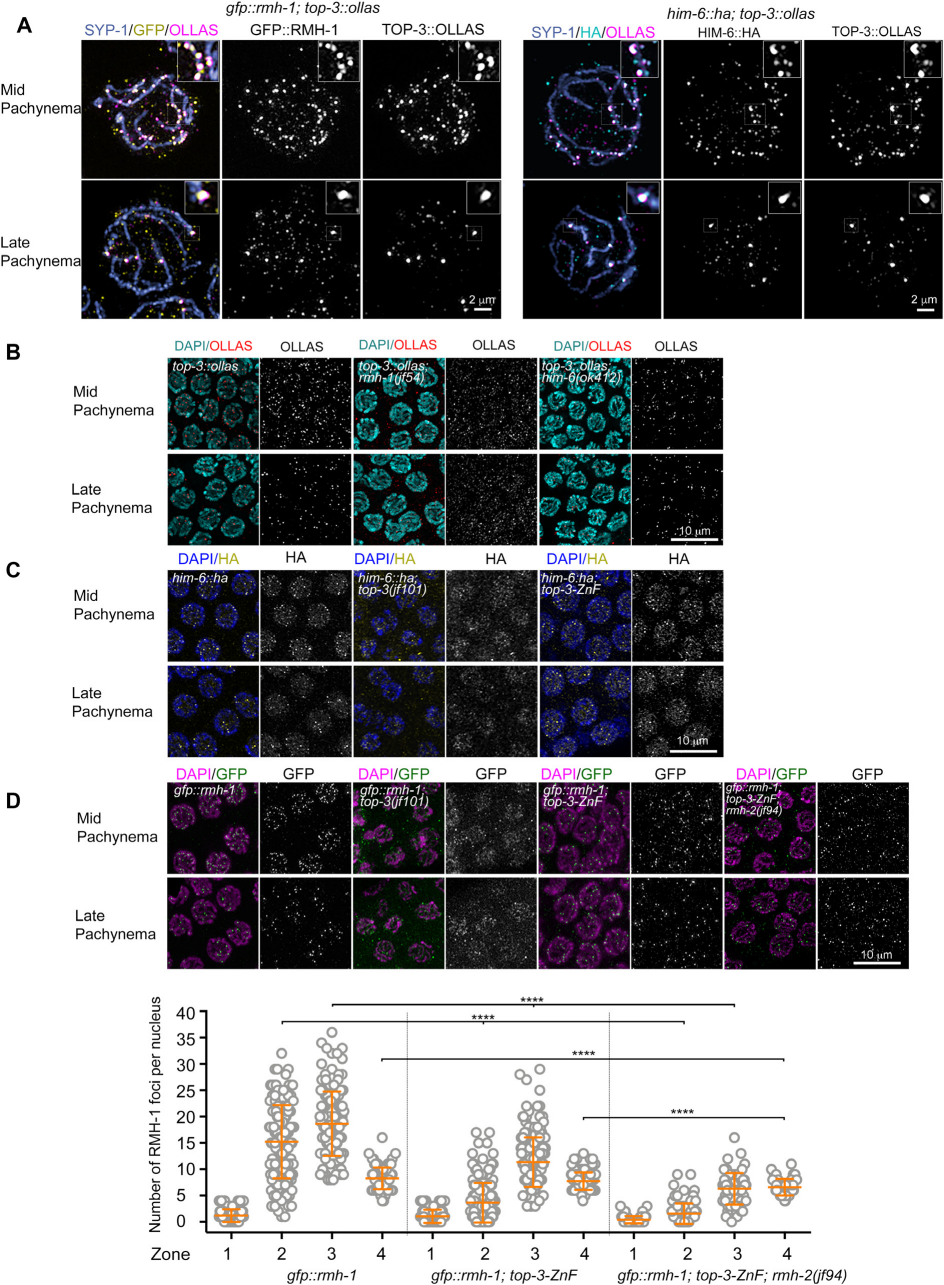
The TOP-3-ZnF domain influences localization of the BTR complex. (**A**) Super-resolution images of *gfp::rmh-1; top-3::ollas* (left) and *him-6::ha; top-3::ollas* (right) nuclei in mid pachynema (top) and late pachynema (bottom). Nuclei were stained with anti-SYP-1 (blue), anti-GFP (yellow) and anti-OLLAS (magenta) antibodies (left) and with anti-SYP-1 (blue), anti-HA (cyan) and anti-OLLAS (magenta) antibodies (right). Half part of the nucleus was projected. Insets (top, right) show magnified boxed regions, highlighting co-localization of the proteins. (**B**) TOP-3::OLLAS localization in mid pachynema (top) and late pachynema (bottom) in the indicated genotypes. Gonads were stained with DAPI (cyan) and anti-OLLAS antibody (red). (**C**) HIM-6::HA localization in mid pachynema (top) and late pachynema (bottom) in the indicated genotypes. Gonads were stained with DAPI (blue) and anti-HA antibody (yellow). (**D**) Upper panel: GFP::RMH-1 localization in mid pachynema (top) and late pachynema (bottom) in the indicated genotypes. Gonads were stained with DAPI (magenta) and anti-GFP antibody (green). Lower panel: quantification of GFP::RMH-1 foci in the meiotic region of the *C. elegans* gonad. The scatter plot indicates the number of foci per nucleus: zone 1 = transition zone; zone 2 = early pachynema; zone 3 = mid pachynema; zone 4 = late pachynema. For each genotype, the mean ± SD number of foci in each zone are indicated, with *n* = the number of nuclei assessed. *gfp::rmh-1*: zone 1, 1.2 ± 1.2, *n* = 111; zone 2, 15.22 ± 6.9, *n* = 155; zone 3, 18.64 ± 6.1, *n* = 44; and zone 4, 8.3 ± 2.1, *n* = 92. *gfp::rmh-1; top-3-ZnF*: zone 1, 1.1 ± 1.3, *n* = 103; zone 2, 3.6 ± 3.8, *n* = 167; zone 3, 11.4 ± 4.7, *n* = 186; and zone 4, 7.7 ± 1.7, *n* = 114. *gfp::rmh-1; top-3-ZnF; rmh-2*: zone 1, 0.4 ± 0.7, *n* = 83; zone 2, 1.6 ± 3, *n* = 89; zone 3, 6.3 ± 3, *n* = 62; and zone 4, 6.6 ± 1.6, *n* = 47. **** *P* < 0.0001, as determined using the Mann–Whitney test; non-significant differences are not shown. (See Supplementary table 1 for more details of the statistical analysis).

We used the OLLAS-tagged TOP-3 strain (16) in co-localization analyses with other BTR complex proteins. Both GFP::RMH-1 and HIM-6::HA localize to dynamic foci throughout pachynema, with the number of foci peaking in mid pachynema and reducing to around six foci per nucleus by late pachynema (16,21). High-resolution imaging revealed that TOP-3 foci mostly co-localize with RMH-1 and HIM-6 on early and late recombination intermediates (Figure 4A).

We investigated the interdependency of BTR complex subunits for DNA association by analyzing TOP-3::OLLAS localization in *rmh-1* and *him-6* mutants and GFP::RMH-1 and HIM-6::HA localization in the *top-3(jf101)* disruption allele. We observed that in *rmh-1*, TOP-3::OLLAS was detected not as discrete foci but as a diffuse signal in the gonad, with sporadic faint foci, indicating that RMH-1 is necessary to concentrate TOP-3 into foci (Figure 4B). This result was confirmed in a reciprocal analysis: in *top-3(jf101)*, GFP::RMH-1 localized as a diffuse signal in the gonad, with some faint foci, suggesting interdependent localization of RMH-1 and TOP-3 (Figure 4D).

Assessment of TOP-3::OLLAS localization in the *him-6* mutant revealed that, as observed for RMH-1 (21), *him-6* is required for TOP-3 localization in early pachynema but not in mid–late pachynema (Figure 4B). In *him-6; top-3::ollas*, few faint TOP-3::OLLAS foci were detected in mid pachynema, whereas in late pachynema the number of TOP-3::OLLAS foci was limited to almost six per nucleus (similar to in *top-3::ollas*). In a reciprocal analysis, we examined the HIM-6::HA localization in *top-3(jf101)*. Similar to GFP::RMH-1, HIM-6::HA appeared as a diffuse signal in the gonad, along with a few faint foci, suggesting that TOP-3 is necessary to concentrate HIM-6 into foci on recombination intermediates (Figure 4C). Taken together, these data indicate that TOP-3 is needed for the correct localization of RMH-1 and HIM-6 to DNA-associated foci.

Next, we examined localization of HIM-6::HA and GFP::RMH-1 in the *top-3-ZnF* mutant to examine how removal of the ZnF domain affects the dynamics of BTR foci appearance throughout pachynema. In *top-3-ZnF*, both HIM-6::HA and GFP::RMH-1 were concentrated into foci with the same dynamics observed in *top-3-ZnF::ha*: early foci were delayed and reduced in number, whereas late foci were unaffected by absence of the TOP-3 ZnF domain (Figure 4C and D).

Quantification of GFP::RMH-1 foci revealed an average of 15.2 (±6.9 SD) foci per nucleus in *gfp::rmh-1* and of 3.6 (± 3.8 SD) in *gfp::rmh-1; top-3-ZnF* in zone 2 (corresponding to early pachynema), with a peak number of foci per nucleus in zone 3 (mid pachynema) in both strains; however, in the absence of the ZnF domain, the peak number of foci per nucleus was reduced by around half *(gfp::rmh-1*, 18.6 (±6.1 SD); *gfp::rmh-1; top-3-ZnF*, 11.4 (± 4.7 SD)). In contrast, in zone 4, no significant difference between the two genotypes was observed (Figure 4D). These results indicate that removal of the ZnF domain might affect the concentration of BTR complex proteins into foci on early recombination intermediates or how these early intermediates are processed before being recognized by BTR complex proteins.

### The TOP-3 ZnF domain is needed to recruit and/or stabilize MSH-5 on early recombination intermediates

MSH-5 and COSA-1 are involved in establishing inter-homolog COs (49–51). MSH-5 localization dynamics are similar to those of BTR complex proteins and MSH-5 partially co-localizes with RMH-1 (21,52,53).

**Figure 5.**
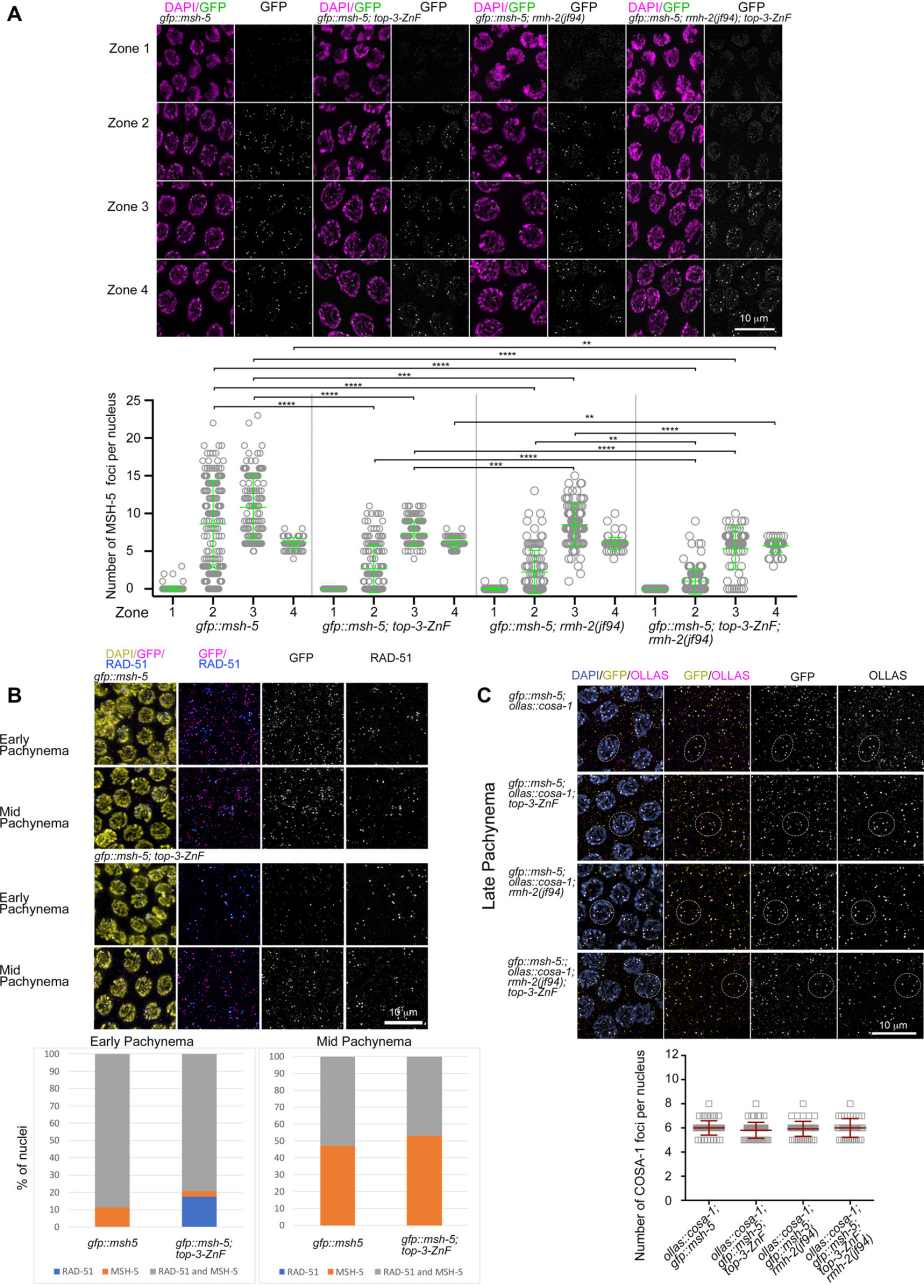
Reduced loading of MSH-5 into recombination foci in *top-3-ZnF* and *rmh-2*. (**A**) Upper panel: representative images of GFP::MSH-5 foci localization at different stages of meiotic prophase I in *C. elegans* gonads of the indicated genotypes. Zone 1 = transition zone; zone 2 = early pachynema; zone 3 = mid pachynema; zone 4 = late pachynema. Images of gonads show DAPI staining (magenta) and endogenous GFP (green). Bottom: Quantification of GFP::MSH-5 foci in the meiotic part of the gonad. Scatter plots indicate the number of foci per nucleus. For each genotype, the mean ± SD number of foci in each zone are indicated, with *n* = number of nuclei assessed. *gfp::msh-5*: zone 1, 0.05 ± 0.3, *n* = 157; zone 2, 8.6 ± 5.8, *n* = 149; zone 3, 10.82 ± 4.2, *n* = 122; and zone 4, 6.1 ± 0.7, *n* = 92. *gfp::msh-5; top-3-ZnF*: zone 1, 0.0 ± 0.0, *n* = 151; zone 2, 2.7 ± 3.2, *n* = 135; zone 3, 7.3 ± 1.6, *n* = 133; and zone 4, 6.1 ± 0.7, *n* = 72. *gfp::msh-5; rmh-2(jf94)*: zone 1, 0.02 ± 0.1, *n* = 125; zone 2, 2.2 ± 2.9, *n* = 107; zone 3, 8.5 ± 2.8, *n* = 93; and zone 4, 6 ± 0.8, *n* = 84. *gfp::msh-5*; *top-3-ZnF; rmh-2(jf94)*: zone 1, 0 ± 0, *n* = 120; zone 2, 1 ± 1.7, *n* = 110; zone 3, 5.3 ± 2.7, *n* = 70; and zone 4, 5.7 ± 0.8, *n* = 58. Statistical significance was determined using the Mann–Whitney test (see Supplementary table 1 for more details of the statistical analysis). (**B**) Upper panel: representative images of nuclei in early and mid pachynema in the indicated genotypes after staining for RAD-51 (blue), GFP (magenta) and DAPI (yellow). Lower panel: the percentage of nuclei in early and mid pachynema that were positive for the indicated meiotic markers (RAD-51 only, blue; MSH-5 only, orange; and both RAD-51 and MSH-5, gray). (**C**) Upper panel: representative images of GFP::MSH-5 and OLLAS::COSA-1 co-localization in recombination foci in late pachynema in the indicated genotypes. Gonads were stained with DAPI (blue), anti-GFP (yellow) and anti-OLLAS (magenta) antibodies. Dashed circles highlight nuclei with clear co-localization. Lower panel: quantification of OLLAS::COSA-1 foci in late pachynema in the indicated genotypes. The scatter plot indicates the number of foci per nucleus. Mean ± SD are indicated, *n* = number of nuclei assessed. *ollas::cosa-1; gfp::msh-5*, 6 ± 0.6, n = 63; *ollas::cosa-1; gfp::msh-5; top-3-ZnF*, 5.8 ± 0.7, *n* = 67; *ollas::cosa-1; gfp::msh-5; rmh-2*, 5.9 ± 0.6, *n* = 52; *ollas::cosa-1; gfp::msh-5; top-3-ZnF; rmh-2*, 6 ± 0.8, *n* = 41. There is no significant difference between the indicated genotypes. Statistical significance was determined using the Mann–Whitney test.

To understand whether delayed RMH-1 and HIM-6 loading onto recombination intermediates is associated with a similar delay in MSH-5 foci appearance, we examined MSH-5 foci dynamics in *gfp::msh-5; top-3-ZnF*. Indeed, GFP::MSH-5 localization was also reduced and delayed when TOP-3 lacks the ZnF domain (Figure 5A). In zone 2, there were an average of 8.6 (± 5.8 SD) foci per nucleus in *gfp::msh-5*, but only 2.3 (±2.8 SD) foci per nucleus were present in *gfp::msh-5; top-3-ZnF*. Similarly, in zone 3, 10.8 (± 4.2 SD) foci per nucleus were present in *gfp::msh-5*, but only 7.3 (±1.6 SD) *gfp::msh-5; top-3-ZnF*. As previously shown for *gfp::rmh-1*; *top-3-ZnF*, *him-6::ha*; *top-3-ZnF* and *top-3-ZnF::ha*, the average number of foci in zone 4 were similar in the *top-3-ZnF* mutant and wt. Taken together, these data suggest that the ZnF domain of TOP-3 influences the timing of appearance and number of early recombination foci marked by MSH-5 and BTR complex proteins. Indeed, the delayed appearance of foci may be associated with a delay in generating these recombination intermediates. We co-immunostained for RAD-51, a marker of strand invasion, and GFP::MSH-5, a pro-CO factor that stabilizes and protects early meiotic intermediates to support the maturation of COs. We could discriminate between nuclei displaying only one of these markers and those displaying both. In early pachynema in *gfp::msh-5*, 88.7% of nuclei contained foci marked with both MSH-5 and RAD-51 and 11.3% contained foci marked with MSH-5 only. In contrast, in early pachynema in *gfp::msh-5; top-3-ZnF*, 79.2% of nuclei contained foci marked with both MSH-5 and RAD-51, 17.5% contained foci marked with RAD-51 only and 3.3% contained foci marked with MSH-5 only (Figure 5B). The presence in *top-3-ZnF* of nuclei containing RAD-51-only foci may indicate that meiotic recombination intermediates were formed but did not mature to the stage of loading the BTR/MSH-5 proteins.

Late recombination intermediates were not affected by removal of the TOP-3 ZnF domain. At that stage, the number of GFP::MSH-5 foci was around six per nucleus.

COSA-1 is another protein detected on maturing and late recombination intermediates. It is a cyclin CNTD1 that in wt is present on the six obligate COs in late pachynema (51). In co-localization analyses using *ollas::cosa-1* (53) and *gfp::msh-5* (Figure 5C), in zone 4 (corresponding to late pachynema) on average six OLLAS::COSA-1 foci were detected in each nucleus (wt, 6 (± 0.6 SD); *top-3-ZnF*, 5.8 (± 0.6 SD)), mostly co-localizing with GFP::MSH-5. This confirms that the late intermediates seen in *top-3-ZnF* are associated with CO markers.

### *rmh-2* mutants resemble *top-3-ZnF* mutants

The *C. elegans* genome encodes two homologs of RMI1: *rmh-1* and *rmh-2*. Although distinct activities have been assigned to RMH-1 in the gonad (16,21), the role of *rmh-2* in the germline has remained undefined. The double mutant *rmh-1; rmh-2* exhibits embryonic death (21).

**Figure 6.**
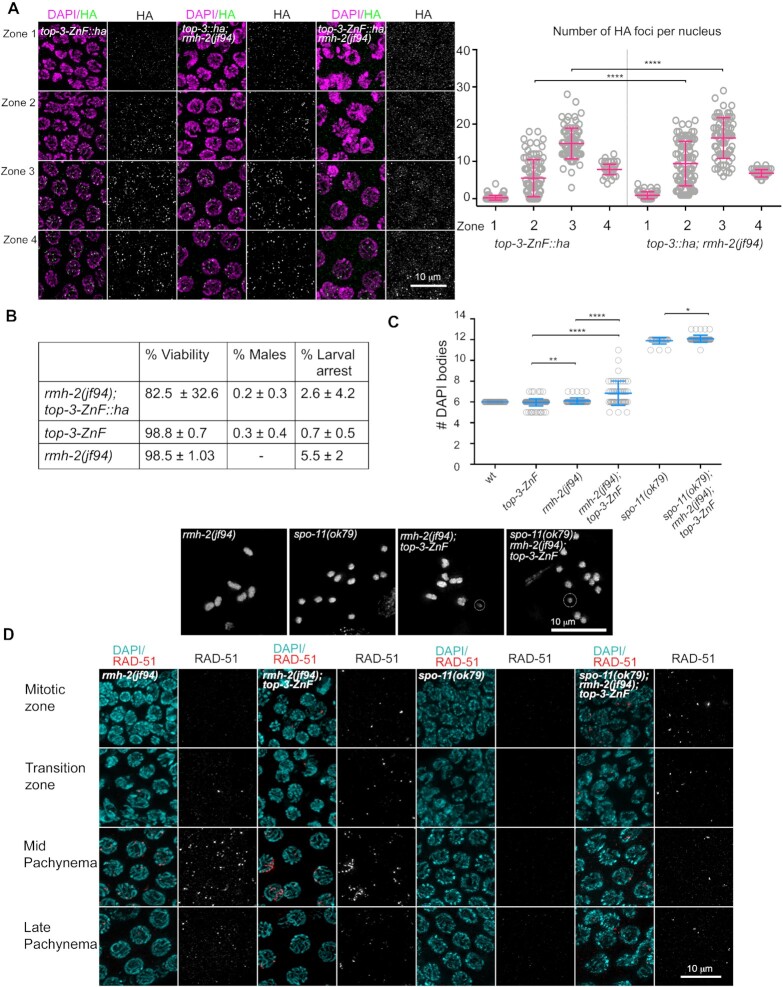
*top-3-ZnF* and *rmh-2* double mutants display a synthetic phenotype. (**A**) Representative images of gonads stained with HA (green) and DAPI (magenta) in the indicated genotypes. Zone 1 = transition zone; zone 2 = early pachynema; zone 3 = mid pachynema; zone 4 = late pachynema. Right panel: HA foci quantification in the indicated genotypes. The scatter plot indicates the number of foci per nucleus. Mean ± SD are indicated, *n* = number of nuclei assessed. *top-3-ZnF::ha* (same quantification as in Figure 3A): zone 1, 0.3 ± 0.6, *n* = 121; zone 2, 5.5 ± 5, *n* = 92; zone 3, 14.8 ± 4.1, *n* = 83; and zone 4, 7.8 ± 1.4, *n* = 63. *top-3::ha; rmh-2*: zone 1, 0.9 ± 0.9, *n* = 89; zone 2, 9.4 ± 6, *n* = 88; zone 3, 16.3 ± 5.4, *n* = 70; and zone 4, 7.4 ± 1.4, *n* = 48. **** *P* < 0.0001, as determined using the Mann–Whitney test; non-significant differences are not shown. *top-3-Zn; rmh-2*: no quantification owing to a lack of foci. (**B**) Percentage viability and rates of larval arrest and males in the indicated genotypes, with *n* = number of worms quantified per genotype: *rmh-2(jf94); top-3-ZnF::han* = 10; and *top-3-ZnF* = 17 (same as in Figure 1C); *rmh-2(jf94)n* = 9. (**C**) Quantification of DAPI bodies in the −1 diakinesis oocyte in the indicated genotypes: wt (same quantification as in Figure 1E) 6 ± 0, *n* = 50; *top-3-ZnF*, 5.96 ± 0.3, *n* = 154 (same quantification as in Figure 1E); *rmh-2*, 6.1 ± 0.3, *n* = 50; *rmh-2; top-3-ZnF*, 6.8 ± 1.2, *n* = 51; *spo-11*, 11.9 ± 0.3, *n* = 28; and *spo-11; rmh-2; top-3-ZnF*, 12.1 ± 0.3, *n* = 49. ** *P* = 0.0099, * *P* = 0.028, **** *P* < 0.0001, as determined using the Mann–Whitney test; non-significant differences are not shown. Insets show representative images of DAPI-stained diakinesis nuclei of the indicated genotypes. The dashed circles highlight fragments of DNA. (**D**) Representative images of RAD-51 foci localization at different stages of meiotic prophase I in the indicated genotypes. Gonads were stained with DAPI (cyan) and anti-RAD-51 antibodies (red).

To assess whether *rmh-2* function might be required for loading and/or stabilizing TOP-3 and pro-CO factors, we analyzed GFP::MSH-5 localization in the *rmh-2(jf94)* mutant. The profile of GFP::MSH-5 foci dynamics in *rmh-2(jf94)* resembled the profile of *top-3-ZnF* (Figure 5A). In *rmh-2(jf94)*, the average number of GFP::MSH-5 foci per nucleus was 1.8 (±2.3 SD) in early pachynema (zone 2), similar to in *top-3-ZnF* (2.3 (±2.8 SD)). In zone 3, the number of foci per nucleus differed significantly between the two mutants, at 7.3 (±1.6 SD) for *top-3-ZnF* and 8.5 (±2.8 SD) for *rmh-2(jf94)*, but was lower in both than in the wt, (*gfp::msh-5* (10.8 (±4.2SD)). In zone 4, six putative CO-marking foci per nucleus were seen in both mutants (Figure 5A), meaning that *rmh-2(jf94)* (like *top-3-ZnF*) is dispensable for GFP::MSH-5 localization to late recombination intermediates. Moreover, in late pachynema GFP::MSH-5 co-localized with OLLAS::COSA-1 in *rmh-2* mutants (5.9 (±0.6 SD) foci per nucleus), similar to in the wt (Figure 5C). Furthermore, in *rmh-2(jf94)*, TOP-3::HA localization to foci was delayed in pachynema, similar to our observations for *top-3-ZnF::ha* (Figure 6A). In zone 2, *rmh-2(jf94)* displayed an average of 9.4 (±6 SD) TOP-3::HA foci per nucleus, which is significantly higher than the average of 5.5 (±5 SD) foci per nucleus observed in *top-3-ZnF::ha* but lower than in *top-3::ha* (14.7 (±7.8 SD) foci per nucleus). In zones 3 and 4, the average number of foci was similar in *rmh-2(jf94)*; *top-3::ha* and *top-3-ZnF::ha*: 16.3 (±5.4 SD) and 14.8 (±4.1 SD), respectively, in zone 3; and 4: 7.4 (±1.4 SD) and 7.9 (±1.4 SD), respectively, in zone 4 (Figure 6A). In summary, these data suggest that RMH-2 might cooperate with the TOP-3 ZnF domain in localizing BTR complex proteins or processing early recombination intermediates.

**Figure 7.**
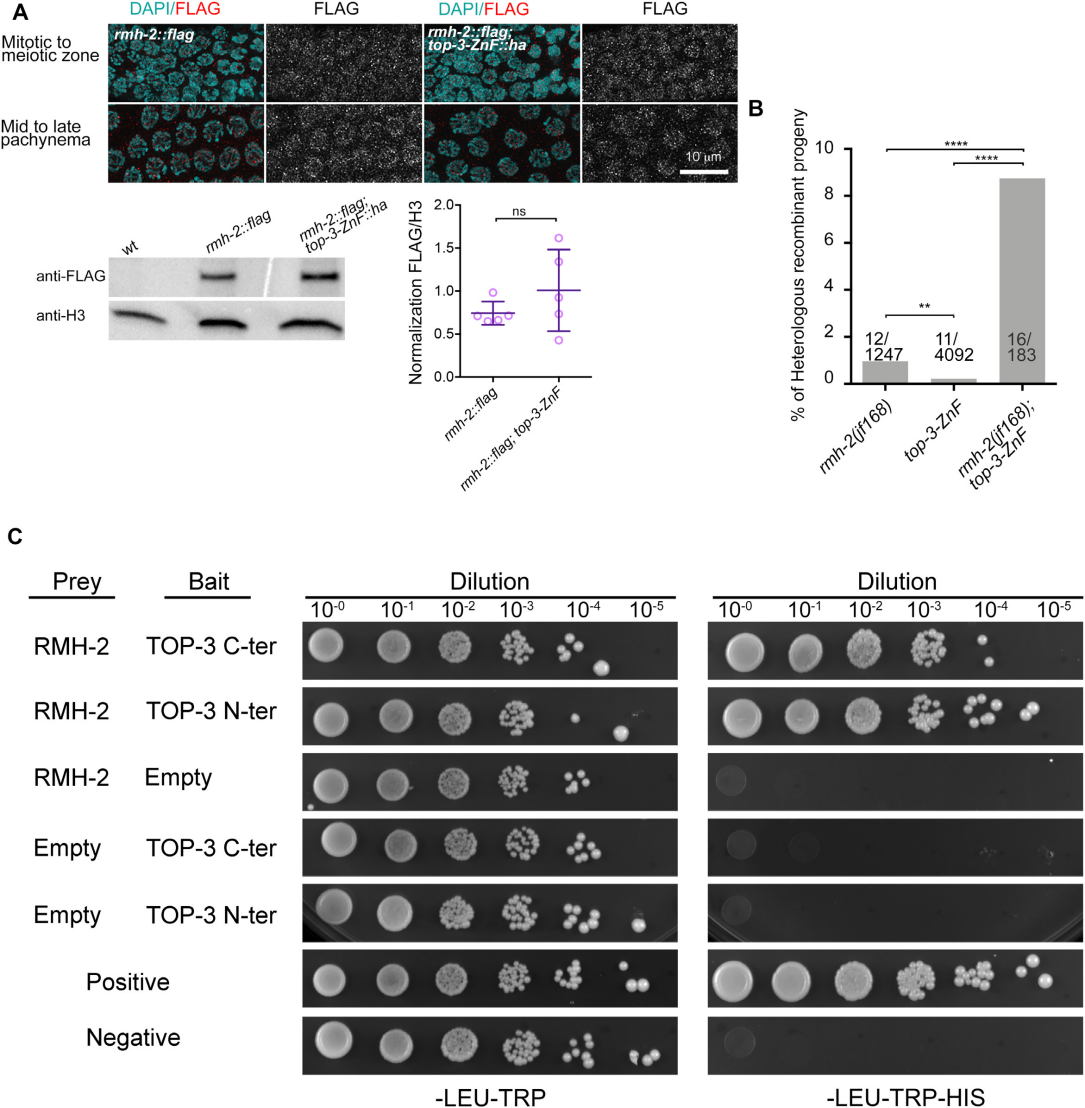
TOP-3 and RMH-2 interact physically and at the genetic level. (**A**) Upper panel: representative images of RMH-2::FLAG in the indicated germline stages, in the absence or presence of *top-3-ZnF*. Lower panel: western blot analysis using anti-FLAG antibodies to detect RMH-2::FLAG in wt and *top-3-ZnF* whole worm extracts. wt worms were used to control for antibody specificity. RMH-2::FLAG is around 100 kDa in size. Histone H3 was the loading control. The scatter plot shows normalized RMH-2::FLAG expression. Five biological replicates were measured for each sample. Error bars indicate the mean and SD. Statistical significance was determined using the Mann–Whitney test. (**B**) Percentage of progeny with heterologous recombination in the *mln1* inversion interval on chromosome 2; the number of recombination events/total number of worms assessed are shown for each genotype. ** P = 0.003; **** *P* < 0.0001 as determined using Fisher's exact test. (**C**) Yeast two-hybrid analysis of interactions between TOP-3 and RMH-2 proteins. As indicated, each spot corresponds to a single dilution. Control plates (SC-Leu-Trp) are on the left and selection plates (SC-Leu-Trp-His) are on the right. Auto-activation of both bait and prey vectors was tested. C-ter: C-terminus; N-ter: N-terminus.

As *top-3-ZnF* mutants have elevated HR frequencies, we wanted to determine whether the same is true for *rmh-2*. Using recombination assays (with the same chromosome intervals described in Figure 2), we examined recombination frequencies and the position and numbers of COs in a new *rmh-2* allele generate by CRISPR/Cas9, *jf168*, where the entire locus was deleted, which behaves identical to *rmh-2(jf94*), (see Supplementary figure S5A for schematic representation of the two *rmh-2* alleles). In fact, no significant different was observed between the hatch rates of *jf168* (95.9% (±6.9 SD)) and *jf94* (98.2% (±1 SD)) as well as for the percentage of larval arrests *jf168* (4.6% (±3.3 SD)) and *jf94* (4.1% (±1 SD)). In *rmh-2(jf168)*, recombination rates were similar to in the wt, but significantly different from in *top-3-ZnF*, and the number and position of COs was similar to in the wt (Figure 2A and Supplementary Figure S3A). Furthermore, the rate of heterologous recombination was 0.8% (8 out of 999 events) in the *rmh-2(jf94*) mutant (Figure 2B and Supplementary Figure S3B) (not different from 0.96% (12 out of 1247) in *rmh-2(jf168*) (Figure 7B and Supplementary Figure S3B)): this is higher than in the wt, similar to in *top-3-ZnF* (0.2%), but significantly lower than in *rmh-1* and *him-6* (∼7%). Taken together, these data indicate that *rmh-2* mutants have phenotypic similarities to *top-3-ZnF*, but with specific differences related to recombination.

### Simultaneous removal of *rmh-2* and *top-3-ZnF* cause synthetic phenotypes

To investigate possible cooperation between TOP-3-ZnF and RMH-2, we generated the *rmh-2(jf94); top-3-ZnF::ha* double mutant and analyzed its phenotype. This mutant had a significantly different hatch rate (82.5% (±32.6 SD)) from the two single mutants: *rmh-2(jf94)*, 98.5% (±1.03 SD); and *top-3-ZnF* 98.8% (±0.7 SD). The rate of male offspring (0.2% (±0.3 SD)) was similar to the rate in *top-3-ZnF* (0.3% (±0.4 SD)), but male offspring were not observed in *rmh-2(jf94)*. The rate of larval arrest (2.6% (±4.2 SD) was lower than in *rmh-2(jf94)* (5.5% (±2 SD)) and *top-3-ZnF* (0.7% (±0.5 SD); Figure 6B).

We next quantified the bivalents at the diakinesis stage to evaluate completion of meiotic prophase I events. *rmh-2(jf92); top-3-ZnF::ha* had an average of 6.8 (±1.2 SD) DAPI-stained bodies, along with chromosome fragments and univalents; this was significantly different in the single mutants, at 6.1 (±0.3 SD) for *rmh-2(jf94)* and 5.96 (±0.3 SD) for *top-3-ZnF* (Figure 6C).

Some of the fragments observed in *rmh-2(jf92); top-3-ZnF::ha* appeared to be independent of meiotic DSB formation since we also found them in the triple mutant *rmh-2(jf92); top-3-ZnF::ha; spo-11(ok79*) (12.1 (±0.3 SD). Figure 6C shows the low level of *spo-11*-independent fragmentation.

Analysis of the dynamics of RAD-51 foci localization in the *rmh-2(jf92); top-3-ZnF::ha* germline revealed defects in DSB processing that were not present in the single mutants (Figure 6D and Supplementary Figure S6A). Chromatin-associated RAD-51 foci were detected in the progenitor cell zone. We observed a peak in foci numbers per nucleus in early–mid pachynema, often appearing as RAD-51 stretches (defined as many foci close to each other that cannot be resolved into single foci). By late pachynema, the number of RAD-51 foci decreased, indicating that some intermediates had eventually been repaired. RAD-51 foci formation was not entirely suppressed upon SPO-11 removal, suggesting that a fraction of the accumulated RAD-51 foci derived from DNA lesions introduced in the progenitor cell zone of the gonad (Figure 6D and Supplementary Figure S6A).

Taken together, these data indicate that *rmh-2(jf92); top-3-ZnF::ha* has a synthetic phenotype that involves defects in processing both mitotic and meiotic DNA lesions.

This synthetic phenotype was specific for *rmh-2(jf92); top-3-ZnF::ha*, because the *rmh-1(jf54); top-3-ZnF* double mutants displayed the *rmh-1(jf54)* phenotype. There were no differences between *rmh-1(jf54); top-3-ZnF* and *rmh-1(jf54)* in embryonic viability (30.0% (±8.6 SD) and 26.5% (±7.6 SD) respectively) or the rate of male offspring (13. 6% (±8.3 SD) and 14.1% (±4.5 SD) respectively; Supplementary Figure S6B). In addition, the number of bivalents at diakinesis was similar in the double mutant (8.9 (±1.6 SD)) and *rmh-1(jf54)* (8.8 (±1.3 SD); Supplementary Figure S6C). Taken together, these results suggest that synergy between the TOP-3 ZnF domain and RMH-2 is more pronounced than that occurring between the TOP-3 ZnF domain and RMH-1.

### TOP-3-ZnF and RMH-2 have synergistic roles in meiotic recombination

We next considered the possibility that TOP-3-ZnF and RMH-2 cooperate during meiosis. To examine whether localization of these proteins is interdependent, we engineered a 3 × FLAG tag into the 3′ end of RMH-2 (for functionality of the strain, see Supplementary Figure S5B–D). The tagged protein was widely expressed in the gonad, including in the progenitor cell zone, and persisted after pachynema. The signal was enriched in nuclei and rather diffuse, with some interspersed foci (Figure 7A). The localization pattern was the same in the *top-3-ZnF::ha* mutant, indicating that the ZnF domain is dispensable for RMH-2 localization. Western blot analysis of whole worm extracts showed no significant difference in expression levels of RMH-2::FLAG protein in wt versus *top-3-ZnF*; however, expression levels were highly variable in the mutant.

We next crossed *rmh-2(jf94)* into *top-3-ZnF::ha*. Strikingly, in the double mutant in early–mid pachynema, TOP-3-ZnF::HA was not concentrated into foci but instead appeared as a diffuse signal. However, a few foci were still detectable in late pachynema (Figure 6A). This result suggests that RMH-2 is needed for the recruitment of TOP-3 lacking the ZnF domain into recombination foci in early–mid pachynema. To examine whether these two proteins cooperate to stabilize other recombination markers, we assessed the localization of GFP::RMH-1 (Figure 4D) and MSH-5::GFP (Figure 5A) in the *rmh-2(jf92); top-3-ZnF::ha* double mutant. Strikingly, both proteins were not loaded onto early recombination intermediates: only onto the late ones (Figure 4D and Figure 5A). In zone 2, there were an average of 1.6 (±1.9 SD) RMH-1::GFP foci per nucleus, and these foci failed to accumulate to numbers seen in the wt. In zones 3 and 4, there were around six foci per nucleus (zone 3, 6.3 (± 3 SD); zone 4, 6.6 (± 1.6 SD)), representing only late recombination intermediates. Similarly, an average of 0.5 (±0.7 SD) MSH-5::GFP foci per nucleus were present in zone 2, and the numbers did not increase in zones 3 and 4, where we only saw late recombination intermediates decorated with GFP::MSH-5 (zone 3, 5.3 ± 2.7 SD; zone 4, 5.7 (±0.9 SD); Figure 5A). In zone 4, GFP::MSH-5 co-localized with OLLAS::COSA-1 in an average of 6 (±0.8 SD) foci per nucleus (Figure 5C). These results confirm the strong functional cooperation between RMH-2 and the TOP-3 ZnF domain in stabilizing the BTR complex on early recombination intermediated.

We analyzed the heterologous recombination events in the *rmh-2(jf168); top-3-ZnF* double mutant by crossing the *mln1* inversion into the double and scoring the recombinant progeny. In this mutant, the rate of heterologous recombination increased to 8.7% (16 out of 183; Figure 7 and Supplementary Figure S3B), similar to rates in the *rmh-1* and *him-6* mutants (15,16). We suspect that the actual rate of heterologous recombination might even be higher because addition of the *mln1* inversion made the double mutant very sick: hatch rates dropped from 82.5% (±32.6 SD) to 53.2% (±26.2 SD) with *mln1*. Furthermore, addition of the *mln1* inversion increased the rate of larval arrest to 26.3% (±17.2 SD; Supplementary Figure S3C). These results suggest that TOP-3-ZnF and RMH-2 synergize to prevent heterologous recombination, with rates of heterologous recombination in the double mutant comparable to those in the *rmh-1* and *him-6* mutants.

### TOP-3-ZnF and RMH-2 physically interact and form protein complexes

To assess whether RMH-2 and TOP-3 physically interact, we performed yeast two hybrid analysis. Use of RMH-2 as prey and TOP-3 as bait (C-terminally or N-terminally tagged) followed by selection revealed an interaction between these proteins (Figure 7C). We also performed co-immunoprecipitation (co-IP) analysis using a strain expressing both *rmh-2::mCherry* and *top-3::ha*. RMH-2::mCherry displayed a similar localization pattern to RMH-2::FLAG in the germline (Supplementary Figure S5B). *rmh-2::flag* showed wt hatch rates (99.5% (± 0.3 SD; Supplementary Figure S5C)), with lower levels of larval arrests (1.1% (±0.8 SD)) than in the *rmh-2* mutant (4.7% (±1.6 SD)) Supplementary Figure S5D). *rmh-2::mCherry* strains had significantly lower hatch rates (86.3% (±9.1 SD)) than the wt (99.7 ± 0.3%) and *rmh-2* mutants (98.3% (±1 SD); Supplementary Figure S5D. This suggests that the tagged line may not be fully functional or that the mCherry tag is inducing an alteration of the phenotype. We counted the number of DAPI-stained bodies at diakinesis in *rmh-2::mCherry* and found that in both the mutant and the tagged line most diakinesis nuclei had six bivalents, with occasional fragments and univalents (*rmh-2(jf94)*, 6.1 (±0.3 SD); *rmh-2::mCherry*, 6.1 (±0.3 SD); Supplementary Figure S5E). Comparison of diakinesis chromosomes in *rmh-2::mCherry; top-3-ZnF* and *rmh-2(jf94); top-3-ZnF* (Supplementary Figure S5E) showed that the tag reduces protein function, but not to the extent seen in the null mutant (*rmh-2::mCherry; top-3-ZnF*, 6.1 (±0.5 SD); *rmh-2(jf94); top-3-ZnF* 6.8 (±1.2 SD)). Nevertheless, we used this strain to co-immunoprecipitate TOP-3 and RMH-2. TOP-3 (with or without the ZnF domain) co-purified with RMH-2 in reciprocal co-IPs (Supplementary Figure S5F). These results provide further evidence that RMH-2 and TOP-3 can interact and may cooperate in some activities.

## DISCUSSION

The topoisomerase 3 activity of the BTR complex has been studied in numerous organisms ((10,11,17,20,23,25,30,36,54)). Besides the catalytic domain, topoisomerase 3 enzymes contain a less well-conserved C-terminal region comprising a variable number of ZnF domains. *C. elegans* TOP-3 contains only one ZnF of the GRF type, which is also found in *Drosophila, Arabidopsis* and mammals. The ZnF domain is considered dispensable for catalytic activity of the topoisomerase (43). Here, we analyzed the function of the TOP-3 ZnF domain in the *C. elegans* germline.

Given the severe meiotic and mitotic defects of the *top-3(jf101)* gene disruption allele (36), we were surprised to discover that the ZnF truncation allele *top-3-ZnF* has such a mild phenotype, with wt viability levels and most diakinesis nuclei containing six DAPI-stained bodies. Our analysis also suggested an absence of replication defects: RAD-51-marked DNA lesions do not accumulate throughout the germline; and no residual RAD-51 was detected in *top-3-ZnF; spo-11*, in which the diakinesis chromosomes were similar to those of *spo-11* mutants.

TOP-3 localizes to discrete nuclear foci in the germline from the transition zone to late pachynema. Upon removal of the ZnF domain, the protein is still concentrated into foci, although with a slightly different localization pattern. Early TOP-3-containing meiotic recombination foci (seen in zones 2 and 3 in the wt) were delayed and reduced in number, but the number and timing of late foci (in late pachynema) were not different from the wt, for a summary model see Supplemental Figure S7.

Also similar to the wt, BTR complex components and the pro-CO proteins, MSH-5 and COSA-1, were concentrated in DNA-associated foci in the *top-3-ZnF* mutant. Nevertheless, in the absence of the ZnF domain, early recombination intermediates containing BTR complex proteins and MSH-5 were also delayed and reduced in number. Our immunofluorescence data revealed that, although recombination is initiated on time, the processing of intermediates is delayed (Figure 5B) but does not ultimately impede CO formation. Therefore, the TOP-3 ZnF domain seems to be required for interaction of the BTR complex and MSH-5 with early recombination intermediates or for their initial processing.

Similar to the HIM-6 Bloom helicase, but in contrast to ZHP-3/4, a complex needed for CO maturation, the ZnF finger domain of TOP-3 is dispensable for the formation of the putative six foci per nucleus in late pachynema (21,55,56). This further supports the hypothesis that genetically distinct BTR subcomplexes exist, differing in their requirements for association with recombination intermediates (requiring either HIM-6 and/or TOP-3-ZnF or both RMH-1 and ZHP-3/4, with the latter needed for recombination intermediates that are eventually detectable as the six foci in late pachynema).

As previously reported in *Arabidopsis* (25,30), recombination analysis in the *top-3-ZnF* mutant revealed an increased number of double COs compared with the wt, indicating that the ZnF domain is required to prevent the formation of extra COs. The extra COs were not marked with the canonical pro-CO proteins: numbers of COSA-1 or MSH-5 foci in late pachynema in the mutant were the same as in the wt. In contrast to *Arabidopsis*, the *top-3-ZnF* mutant had few joint DNA structures, suggesting that the TOP-3 ZnF domain is required to remove hemicatenates or relieve topological constrains to some degree.

The *top-3-ZnF* mutant was only mildly defective in discouraging wrong or unproductive strand invasions into regions of low sequence identity, which can ultimately be detected as heterologous recombination events and in the wt might be taken care of by D-loop reversion or dissolution. Our results suggest that heterologous recombination does not usually occur in the wt. In contrast, *top-3-ZnF* mutants displayed a very low, but significant different, rate of heterologous recombination, indicating a possible defect in the ejection of unproductive strand invasions. The fact that the rates were lower than in *rmh-1*, *him-6* or *rmif-2* mutants (15,16) might mean that removal of the ZnF domain results in an hypomorphic phenotype regarding stable association of the complex with recombination intermediates. In this mutant, the TOP-3 catalytic domain remains intact and BTR complex association with recombination intermediates may be only slightly compromised.

Phenotypic analysis of the *top-3-ZnF; mus-81* double mutant also suggested the accumulation of aberrant strand invasions (resulting in the formation of aberrant joint molecules). In *Arabidopsis*, in the absence of AtMUS81 the T1 ZnF domain of AtTOP3 is required to resolve joint structures, suggesting that the ZnF domain is involved in recruitment to intermediates (17). This may also be the case in *C. elegans*, as the *top-3-ZnF; mus-81* double mutant has a worse phenotype than the *mus-81* single mutant.

The RTEL-1 helicase works in parallel to BTR-mediated SDSA to dismantle early joint DNA structures. Both *rmh-1* and *him-6* double mutants with *rtel-1* have a severe embryonic death phenotype (21,46). Phenotypic analysis of the *rtel-1; top-3-ZnF* double mutant revealed that it was viable but had a reduced hatch rate, with some larval arrest, indicating less efficient repair of recombination events. Altogether, these findings support the notion that the *top-3-ZnF* mutant is a hypomorph allele of *top-3* that contributes to the removal of joint molecules.

*C. elegans* has two RMI1 homologs: RMH-1 and RMH-2. Gene duplication is not uncommon in this organism, followed by the functional specification of duplicated genes (e.g. (42)). Although much is known about RMH-1(21), RMH-2 activities in the gonad are poorly characterized. Our biochemical and genetic analyses indicate that RMH-2 interacts with TOP-3 to perform some joint activities, possibly as part of a BTR subcomplex. RMH-1 pull-down experiments did not detect an interaction with RMH-2 (16), suggesting that RMH-1 and RMH-2 may not form part of the same complex.

We observed synergy between RMH-2 and the TOP-3 ZnF domain in associating with or processing early recombination intermediates. Strikingly, in the *rmh-2; top-3-ZnF* double mutant only late recombination foci containing RMH-1, MSH-5 or COSA-1 were detected in the gonad. Moreover, possible synergy between RMH-2 and the TOP-3 ZnF domain was strikingly apparent in the *rmh-2; top-3-ZnF* double mutant, which displayed a similar high rate of heterologous recombination as observed in *rmh-1* and *him-6* single mutants. The *rmh-2; top-3-ZnF* double mutant tolerates the introduction of the of *mln1* inversion chromosome very poorly, making the cross difficult to maintain.

*rmh-2* mutants displayed wt levels of COs and recombination events, indicating that (unlike in *top-3-ZnF*) RMH-2 is not required to prevent the formation of extra COs or that an activity of RMH-1 can compensate for the loss of RHM-2 when extra COs are formed.

The subcellular localization of RMH-2 is totally different from other BTR complex proteins (16,21,36,52): in the gonad, RMH-2 appears as a diffuse signal, including cells in the mitotic germ cell compartment until diplonema. RMH-2 is required for TOP-3 concentration into early recombination foci, but lack of TOP-3 does not alter the RMH-2 localization (at least at the resolution level of our immunofluorescence analysis).

Western blot analysis of RMH-2::FLAG whole worm extracts of the *top-3-ZnF; rmh-2::flag* mutant revealed highly variable expression levels, causing large standard deviations that we did not observe in the *rmh-2::flag* mutant. This suggests that RMH-2 protein is less stable in the *top-3-ZnF* mutant background and supports the notion that association between the two proteins is needed for their stable recruitment to recombination intermediates.

The *rmh-2; top-3-ZnF* double mutant exhibited a severe synthetic phenotype with defective processing of both meiotic and mitotic DNA lesions. The *rmh-2; top-3-ZnF* double mutant had reduced viability, with chromosome fragments and univalents seen at diakinesis, although fewer than in the *rmh-1* mutant. These synthetic phenotypes and the fact that RMH-2 and TOP-3 proteins interact and could be co-purified from *in vivo* protein complexes strongly argues for functional cooperation between the proteins.

We speculate that RMH-2 and TOP-3 may also cooperate during mitosis to repair compromised replication forks. Interestingly, the mitotic phenotype of the *rmh-2; top-3-ZnF* double mutant is less severe than that of the *top-3(jf101)* disruption allele, indicating that cooperation of these proteins may be less relevant when the topoisomerase domain is functional in the progenitor zone of the germline.

We conclude that the TOP-3 ZnF domain is required for BTR complex activities at recombination sites and cooperates with RMI1 (in particular, with RMH-2 in *C. elegans*) in promoting the stable association of the BTR complex with recombination intermediates. Cooperation between the TOP-3 ZnF domain and RMH-2 seems to be more important for processing early meiotic recombination foci than for processing DNA lesions in the progenitor cell zone.

## DATA AVAILABILITY

All relevant data are within the manuscript and its supporting files.

## Supplementary Material

gkac408_Supplemental_Files
