# Supplementary material for: The topoisomerase 3 zinc finger domain cooperates with the RMI1 scaffold to promote stable association of the BTR complex to recombination intermediates in the Caenorhabditis elegans germline
Source: Nucleic Acids Res. 2022 May 27;50(10):5652–71. doi: 10.1093/nar/gkac408 (PMC9178014; doi:10.1093/nar/gkac408)
Supplement: gkac408_Supplemental_Files [file gkac408_supplemental_files.zip › Supporting material_revised_4.4_final.pdf]

Supporting material

Supplementary Table 1 excel file: "Statistical analysis”

pp. 2-3 **Supplementary Figure S1** “The ZnF domain is conserved in different topoisomerases*”*

p. 4 **Supplementary Figure S2** “*top-3-ZnF* mutants have a less severe phenotype than *top-3(jf101)”*

p. 5 **Supplementary Figure S3** “*top-3-ZnF* displays extra COs and few heterologous recombination events”

pp. 6–7 **Supplementary Figure S4** “ Chromatin associated TOP-3 in different genotypes”

pp. 8–9 **Supplementary Figure S5** “Strain functionality and co-immunoprecipitation*”*

p. 10 **Supplementary Figure S6** “TOP-3 cooperates with RMH-2 in a different way than with RMH-1*”*

p. 11 **Supplementary Figure S7 “**Summary model highlighting the role of the TOP-3 Zinc Finger domain in complex localization”

pp. 12–13 **Supplementary Materials and Methods**

pp. 14–15 **Strain list**

Supplementary Figure S1. The ZnF domain is conserved in different topoisomerases

A

H. sapiens	Bloom helicase	TOP3	RMI1	RMI2
C. elegans	HIM-6	TOP-3	RMH-1, RMH-2	RMIF-2

B

** jf153/ top-3-ZnF*

```
A. thaliana       --------------ARRTPGT---APSNNIQGSNTRQSNV--CIHCQQ------------
H. sapiens        SLNRMDNSQHPQPADSRQTGSSKALAQTLPPPTAAGESNSVTC-NCGQEAVLLTVRKEGP
C. elegans        ----------PKP-ARKPR-----APRKSAAPKEQEEEEEVFC-----------------
D. melanogaster   SLGTAAPTPPPKPAAKRPRWDS--VERDSTPPSSVPESETVLCTGCQQPARQNTVRKNGP
                                  .               .   :.:   *

A. thaliana       -RGHASTNCPSR------------------------------------------VPASRN
H. sapiens        NRGRQFFKCNGGS-CNFFLWADSPNPGAGGPPALAYRPLGA---------SLGCPPGPGI
C. elegans        -------QCPEP------------------------------------------------
D. melanogaster   NLGRLYYKCPKPDECNFFQWADEPPSSAKSKNSTGSAPQSTTSWGSNRVVTLPSIQQSNS
                         :*

A. thaliana       SRPTATNPRNDESTVSC----------------------NTCGSQCVLRTANTEA-NRGR
H. sapiens        HLGGFGNPGDGSGSGTS----------------------CLCSQPSVTRTVQKDGPNKGR
C. elegans        -------------------------------------------MRAVTKVVQKEGPNKGK
D. melanogaster   QRGQSSMRSNSSSTVTITQTKTKQQERNTATPGDGEEVMCNCGQLASQLTVRKDGPNQGR
                                                               .   ....:. *.*.

A. thaliana       QFFSCP-----TQGCSFFAWEDSINNSSGNATTGSNSGGSGRRGSRGRGRGGRGGQSSGG
H. sapiens        QFHTCAKP--REQQCGFFQWVDENTAPGTSGAPSWTGDRGRTLESEARSKRPRASSSDMG
C. elegans        KFYTCSLPYTSSEKCNFFKWA---------------------------------------
D. melanogaster   PFYACP---TREKSCGFFKWGDEDQNQGASSTS---------WGSANRNPPGRSQPTAIT
                   *.:*.      : *.** *

A. thaliana       RRGSGTSFVSATGEPVSGIRCFSCGDPSHFANACPNRNNSNGNYF
H. sapiens        STAKKPR------------KCSLCHQPGHTRPFCPQNR-------
C. elegans        ---------------------------------------------
D. melanogaster   SDGPKTR------------RCGLCRKEGHTRNKCPRKDEFDM---
```

C

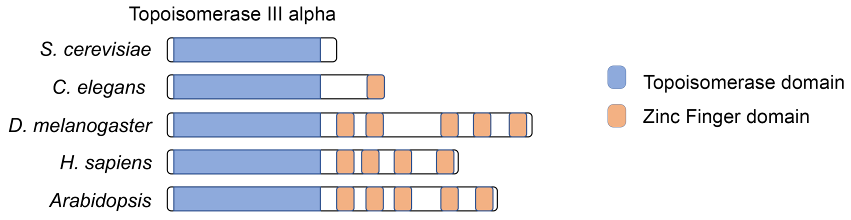

D

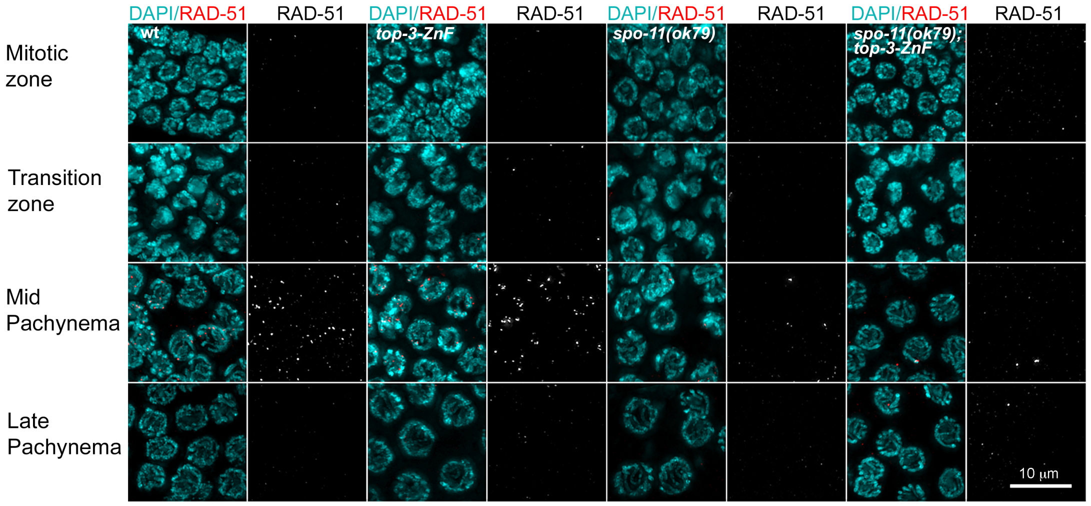

Supplementary Figure S1: The ZnF domain is conserved in different topoisomerases. (**A**) Table with the BTR complex proteins in *C. elegans* and *H. sapiens*. (**B**) Alignment of the topoisomerase 3 GRF zinc finger domain in the indicated species to highlight the evolutionary conservation of the domain,

using the tools described in (1). GRF zinc-coordinating (cysteines) and DNA-binding residues are marked in orange and with an asterisk. (**C**) Schematic representation of various domains in Topoisomerase 3 alpha in the different model systems. (**D**) Representative images of RAD-51 foci localization at different stages of meiotic prophase I in the indicated genotypes. Gonads were stained with DAPI (cyan) and an anti-RAD-51 antibody (red).

Supplementary Figure S2. *top-3-ZnF* mutants have a less severe phenotype than *top-3(jf101)*

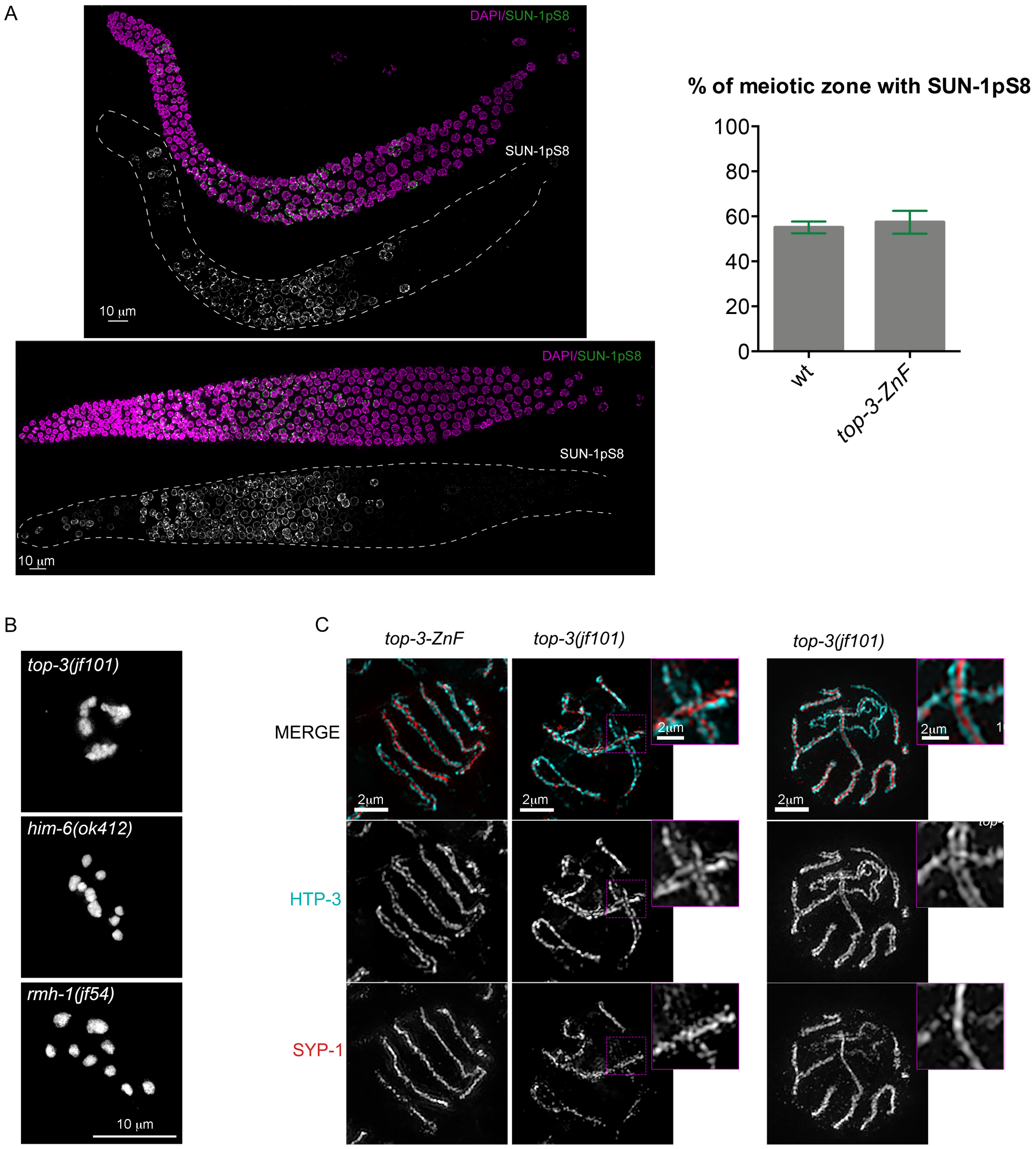

Supplementary Figure S2. *top-3-ZnF* mutants have a less severe phenotype than *top-3(jf101)*. (**A**) Left panel: representative gonad images for the indicated genotypes stained for DAPI (magenta) and SUN-1pS8 (green). Right panel: graph showing quantification of the meiotic zone positive for phospho-SUN-1S8. (**B**) Representative images of DAPI-stained diakinesis nuclei of the indicated genotypes. (**C**) Representative images of high-resolution nuclei stained for HTP-3 (cyan) and SYP-1(red). Only half of the nucleus is projected. Interlocks can be seen in the gene disruption allele of *top-3(jf101),* but are never seen in *top-3-ZnF*. Dashed squares in magenta indicate the position of the insets in the upper-right corner for each nucleus, containing more detailed images of the interlocks.

Supplementary Figure S3. *top-3-ZnF* displays extra COs and few heterologous recombination events

A

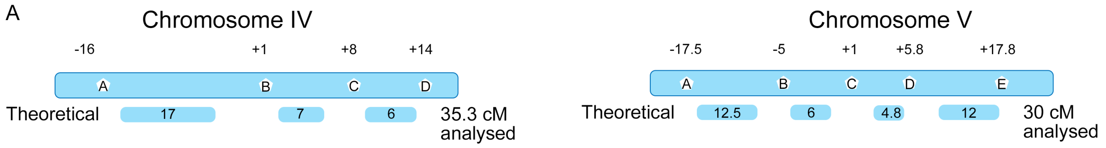

Genetic interval

	AB	BC	CD	n
wt	19.6 (53)	4.4 (12)	5.5 (15)	271
top-3-ZnF	24.1 (66)	9.1 (25)	5.8 (16)	274
rmh-2(jf168)	15 (32)	3.8 (78)	8.4 (18)	213

Genetic interval

	AB	BC	CD	DE	n
wt	11.5 (30)	3.1 (8)	2.3 (6)	18.8 (49)	261
top-3-ZnF	12.5 (34)	11 (30)	7.3 (20)	17.6 (48)	272
rmh-2(jf168)	7.4 (17)	3.5 (8)	3.9 (9)	15.2 (35)	230

B

	Non recombinants			Recombinants				
	**mild-dpy non-rol mild-GFP**	**dpy non-rol non-GFP**	**non-dpy rol bright-GFP**	**dpy non-rol mild-GFP**	**mild-dpy non-rol bright-GFP**	**mild-dpy non-rol non-GFP**	**non-dpy rol mild-GFP**	**Recombinants/** total
**wt**	2275	661	1184	3	0	0	0	**3**/4123 (0.07%)
top-3-ZnF	2192	788	1112	9	1	0	1	**11**/4103 (0.21%)
rmh-2(jf94)	450	237	312	4	0	0	4	**8**/999 (0.8%)
rmh-2(jf168)	711	235	278	5	1	0	6	**12**/1247 (0.9%)
top-3-ZnF; rmh-2(jf168)	96	23	48	3	3	0	9	**16**/183 (8.7%)

C

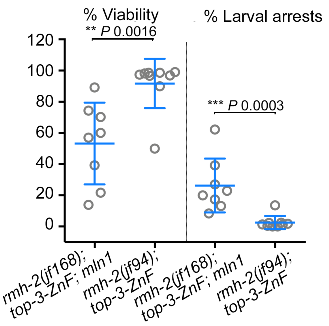

Supplementary Figure S3 *top-3-ZnF* displays extra COs and few heterologous recombination events*.* (**A**) Upper panel: schemes of chromosomes IV and V with the corresponding locations of SNPs used in the PCR-based recombination assay (same as in Figure 2A). Lower panel: tables showing the percentage of recombination in the indicated intervals, with numbers in parenthesis. n = number of worms analyzed. (**B**) Table showing numbers of recombinants scored for the heterologous recombination experiments shown in Fig 2B. Numbers of non-recombinant (gray)/recombinant (light blue) worms are shown for each genotype. (**C**) Scatter plot displaying percentages for embryonic viability and larval arrest in *rmh-2(jf168); top-3-ZnF; mIn1* and *rmh-2(jf94); top-3-ZnF*. *rmh-2(jf168); top-3-ZnF; mIn1*: n = 8, viability = 53.2% ± 26.2%; larval arrest = 26.3% ± 17.2%. *rmh-2(jf94); top-3-ZnF*: n = 9, viability = 91.7% ± 15.9%; larval arrest = 2.6% ± 4.2%. ** *P* = 0.0016; *** *P* = 0.0003, as determined using the Mann–Whitney test.

Supplementary Figure S4. Chromatin associated TOP-3 in different genotypes.

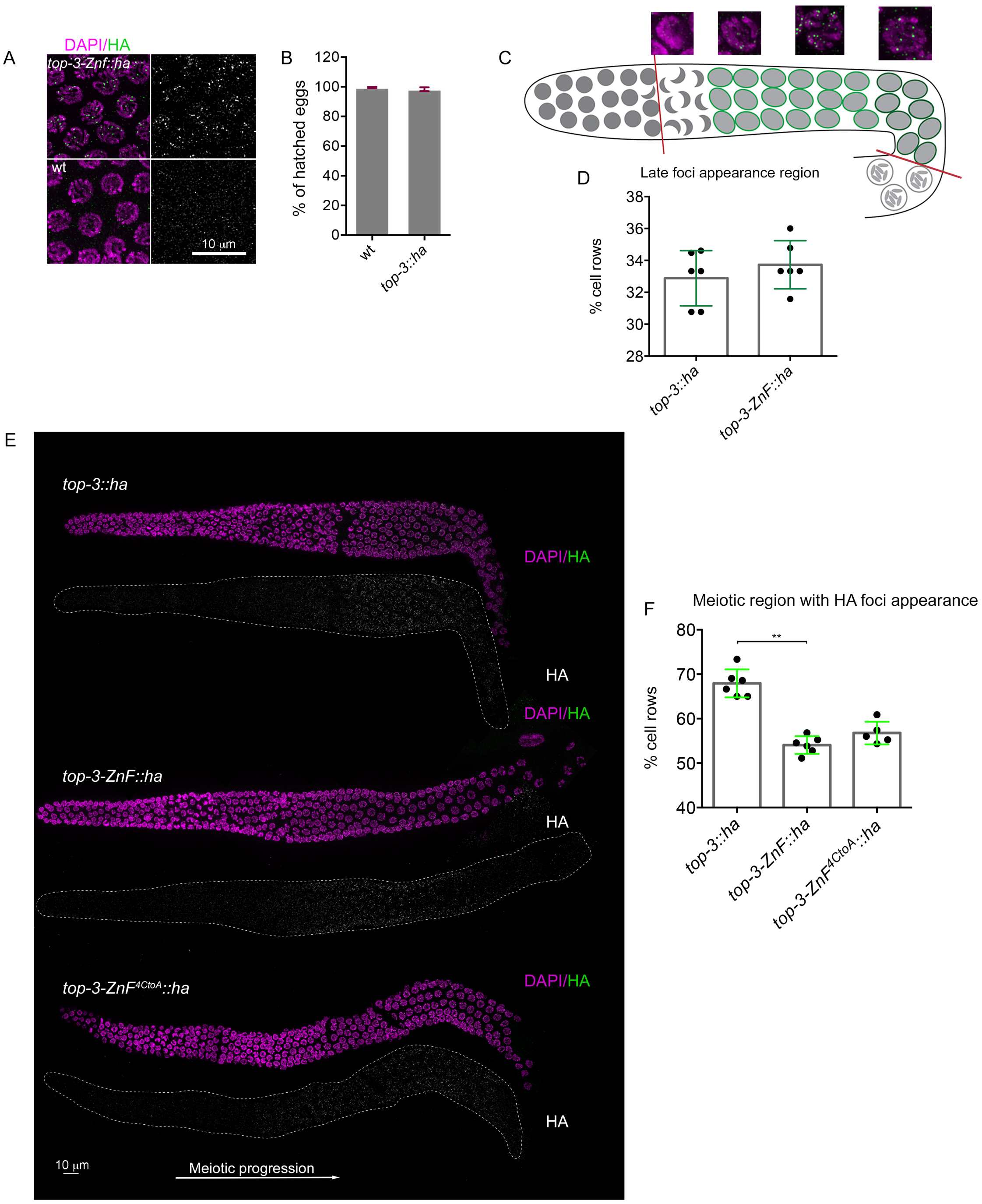

Supplementary Figure S4. Chromatin associated TOP-3 in different genotypes. (**A**) Representative images of *top-3-ZnF::ha* and wt pachynema nuclei stained for DAPI (magenta) and HA (green). (**B**) Chart showing the percentage (mean ± SD) of hatched eggs for the indicated genotypes, with n = number of worms assessed: wt, 99.5 ± 0.4, n = 13; *top-3::ha*, 98.3 ± 1.3, n = 16. Statistical significance was determined using the Mann–Whitney test. ns not shown. (**C**) Schematic representation of a gonad where the red line delineatates the meiotic zone used to quantify the region of late foci appearance (nuclei with dark green borders and the total region positive for HA staining (nuclei with light and dark green borders). (**D**) Graph with dark green error bars displays the region of late foci appearance. Statistical significance was determined using the Mann–Whitney test. ns not shown. (**E**) Representative

images for the indicated genotypes stained for DAPI (magenta) and HA (green). (**F**) Graph with light green error bars shows the quantification of the region positive for HA. Statistical significance was determined using the Mann–Whitney test, ** *P* 0.002.

Supplementary Figure S5. Strain functionality and co-immunoprecipitation

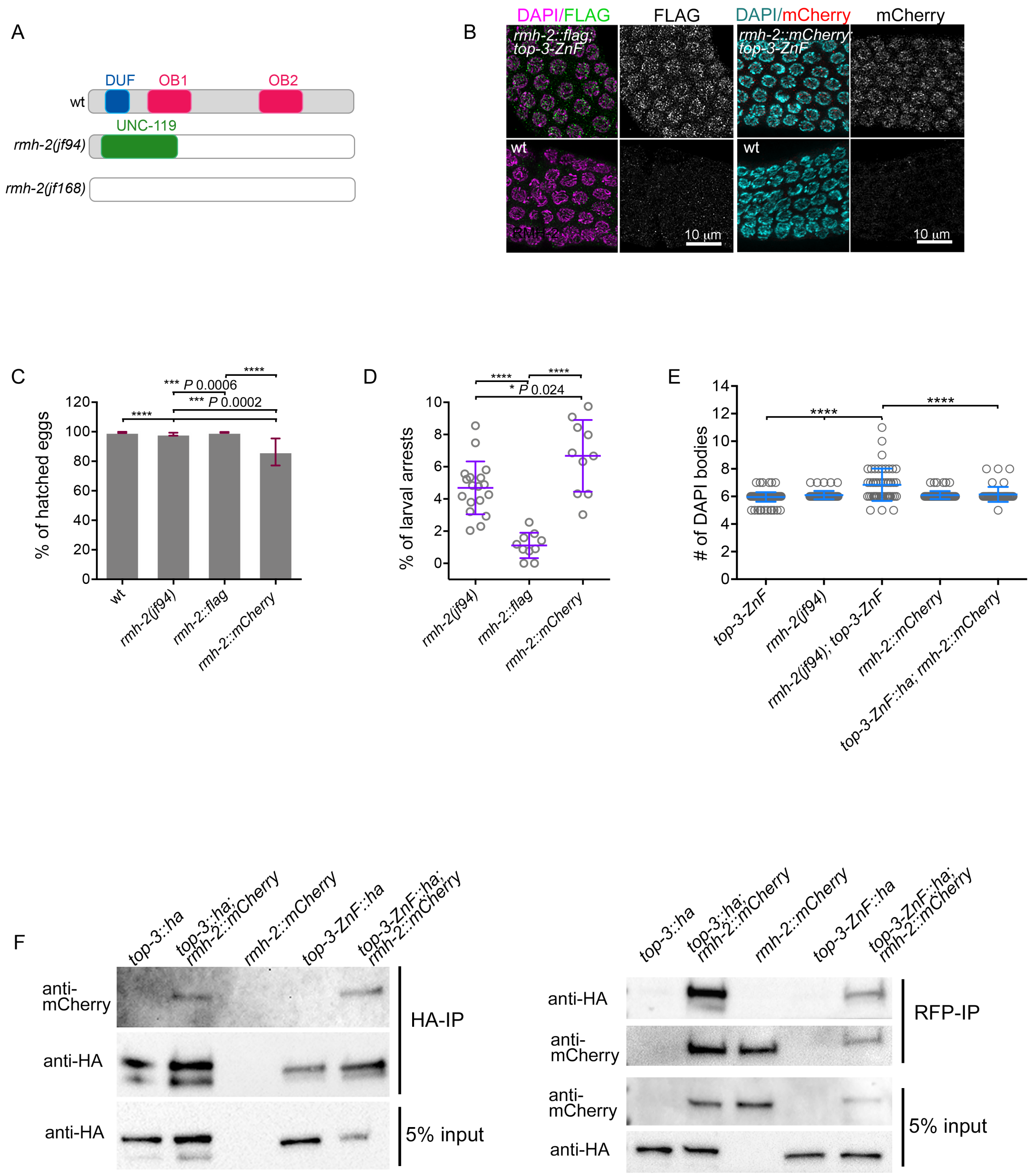

Supplementary Figure S5. Strain functionality and co-immunoprecipitation (**A**) Schematic representation of the two *rmh-2* allele. (**B**) Representative images of *rmh-2::flag; top-3-ZnF* and wt pachynema nuclei stained for DAPI (magenta) and FLAG (green), and *rmh-2::mCherry; top-3-ZnF* and wt pachynema nuclei stained for DAPI (cyan) and mCherry (red). (**C**) Chart showing the percentage (mean ± SD) of hatched eggs for the indicated genotypes, with n = number of worms assessed: wt, 99.5 ± 0.4, n = 13 (same as in Supplementary Figure S4); *rmh-2(jf94)* (same as in Figure 6B), 98.3 ± 1, n = 18; *rmh-2::flag*, 99.5 ± 0.3, n = 10; and *rmh-2::mCherry*, 86.3 ± 9.1, n = 10. Statistical significance was determined using the Mann–Whitney test, **** $P < 0.0001$. (**D**) Scatter plot showing the percentage of larval arrests (mean ± SD) per worm in the indicated genotypes, with n = number of worms assessed: *rmh-2(jf94)* (same as in Figure 6B), 4.7 ± 1.6, n = 18; *rmh-2::flag,* 1.1 ± 0.8, n = 10; and *rmh-2::mCherry*, 6.7 ± 2.2, n = 10. Statistical significance was determined using the Mann–Whitney test, **** $P < 0.0001$.

(**E**) Quantification of DAPI bodies (mean ± SD) in −1 diakinesis oocytes in the indicated genotypes, with n = number of diakinesis oocytes assessed: *top-3-ZnF*, 5.96 ± 0.3, n = 154 (same as in Figure 1E); *rmh-2(jf94)*, 6.1 ± 0.3, n = 50 (same as in Figure 6C); *rmh-2; top-3-ZnF*, 6.8 ± 1.2, n = 51 (same as in Figure 6C); *rmh-2::mCherry,* 6.1 ± 0.3, n = 63; and *rmh-2::mCherry; top-3-ZnF::ha,* 6.1 ± 0.5, n = 48. Statistical significance was determined using the Mann–Whitney test, **** $P < 0.0001$. (**F**) Western blot analysis of HA pull-downs (left panel) and RFP pull-downs (right panel) with the indicated genotypes. The blots were probed with one antibody and, after stripping, with the second antibody. The 5% input sample is shown at the bottom. Biological duplicates were analyzed.

Supplementary Figure S6. TOP-3 cooperates with RMH-2 in a different way than with RMH-1

A

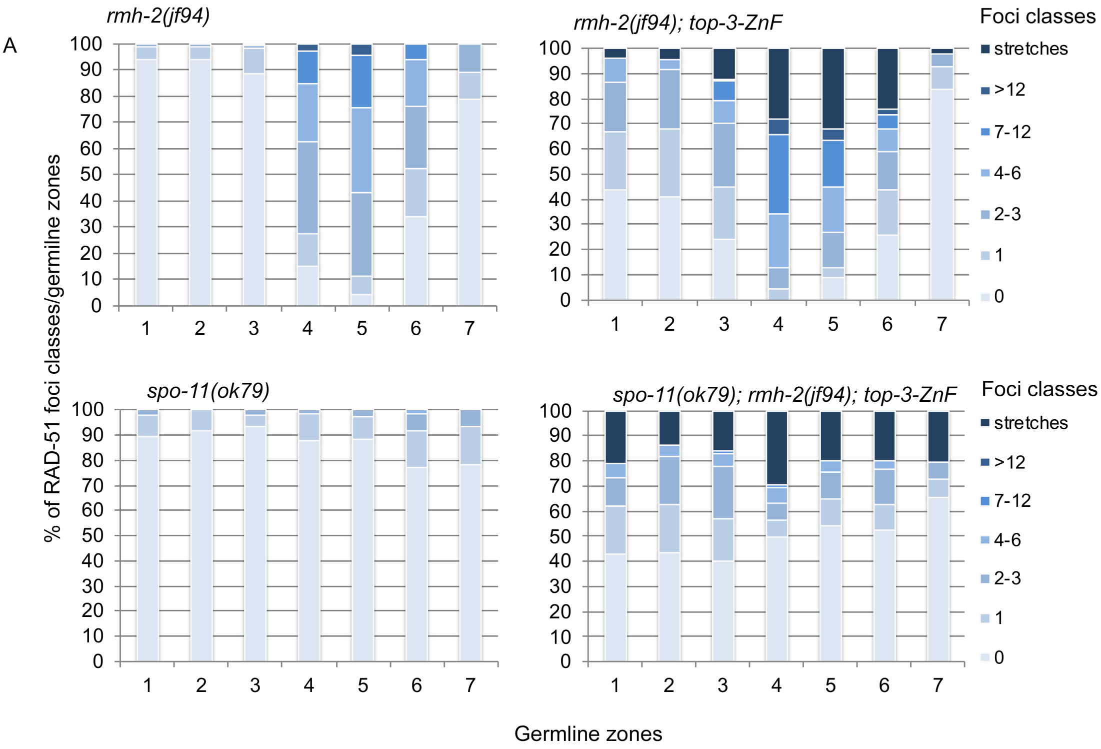

B

	% Viability	% Males
rmh-1(jf54)	26.5 ± 7.6	14.1 ± 4.5
rmh-1(jf54); top-3-ZnF	30.3 ± 8.6	13.6 ± 8.3

C

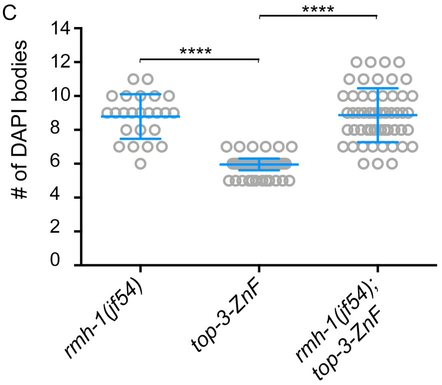

Supplementary Figure S6. TOP-3 cooperates with RMH-2 in a different way than with RMH-1 (**A**) Percentage of RAD-51 foci per nucleus in each of the seven zones in the indicated genotypes. For each zone, the average number of RAD-51 foci per nucleus was calculated from three gonads per genotype. (**B**) Table showing the embryonic viability rate and percentage of males in the indicated genotypes, with n = number of worms assessed per genotype: *rmh-1* n = 9; and *rmh-1; top-3-ZnF* n = 10. (**C**) Quantification of DAPI bodies in −1 diakinesis oocytes (mean ± SD) in the indicated genotypes, with n = the number of diakinesis oocytes assessed per genotype: *rmh-1(jf54)*, 8.8 ± 1.3, n = 24; *top-3-ZnF*, 5.96 ± 0.3, n = 154 (same as in Figure 1E); and *rmh-1(jf54); top-3-ZnF,* 8.9 ± 1.6, n = 54. Statistical significance was determined using the Mann–Whitney test, **** $P < 0.0001$.

Supplementary Figure S7: Summary model highlighting the role of the TOP-3 Zinc Finger domain in complex localization

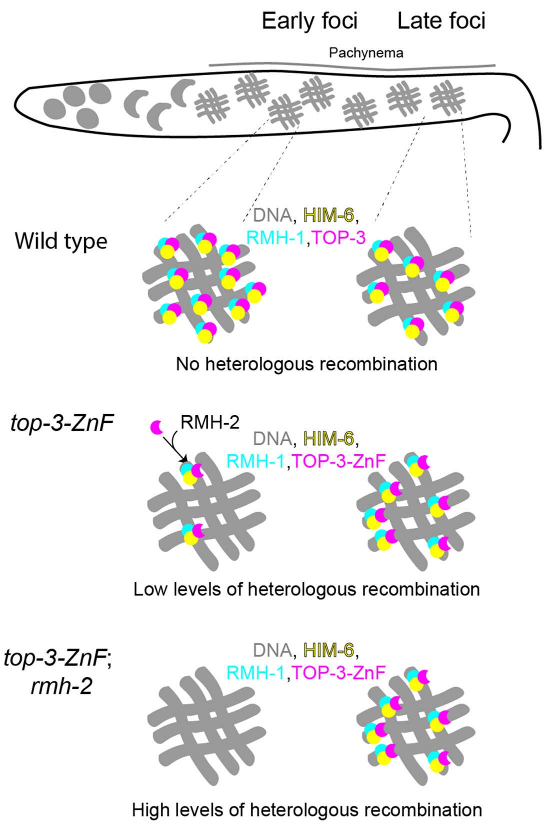

Supplementary Figure S7: Summary model highlighting the role of the TOP-3 Zinc Finger domain in complex localization. Schematic representation of nuclei with early foci (in early/mid-pachynema) and late foci (late pachynema) for the indicated genotypes. In wt, the BTR complex proteins, HIM-6, RMH-1, and TOP-3 are localized in pachynema initially as numerous DNA-associated foci, which are absent in *rmh-2* mutants. Foci of HIM-6, RMH-1, and TOP-3 reduce to around 6 foci per nucleus in late pachynema. In wt, heterologous recombination events are very rare. In absence of the TOP-3 ZnF domain the localization of the early HIM-6, RMH-1, and TOP-3 foci is reduced and delayed. In this genetic background, we observe low levels of heterologous recombination events. In the double mutant *top-3-ZnF; rmh-2* the localization of the complex is limited to late pachynema nuclei. A robust increase in heterologous recombination can be detected. These results highlight the strong functional cooperation between RMH-2 and the TOP-3 ZnF domain in localizing the BTR complex to early meiotic recombination intermediates and in counteracting heterologous recombination.

Supplementary Materials and methods

Image acquisition

Images were acquired using a DeltaVision system equipped with 60×/1.42 and 100×/1.40 oil immersion objective lenses and a DeltaVision Ultra microscope equipped with a 4-megapixel sCMOS camera. Z stacks of 0.20 µm were deconvolved using SoftWoRx software and processed in Adobe Photoshop. Three to five images were acquired using the same settings to cover each gonad. Maximum intensity projections of deconvolved images were generated using Fiji/ImageJ after background subtraction using a rolling ball radius of 50 pixels. Image stitching to build up entire gonads was performed manually in Adobe Photoshop. Relative intensity levels of stitched images were adjusted in Adobe Photoshop to correct for the auto-adjustment settings of the microscope, as previously described (2).

High-resolution images were obtained using a DeltaVision OMX based on the structured illumination method equipped with two sCMOS cameras. Images were obtained as 125-nm spaced Z-stacks using a Plan Apo N 60x/1.42 PSF objective lens. The acquired images were reconstructed and corrected for registration using SoftWoRx before projection using maximum intensity projection in ImageJ.

Quantification and kinetics of RAD-51 *foci*

RAD-51 foci were quantified by dividing the gonad into seven equal zones from the distal gonad tip to late pachynema, and counting the number of foci per nucleus for each zone. The percentage of nuclei with 0 foci, 1 focus, 2–3 foci, 4–6 foci, 7–12 foci, >12 foci and stretches of foci (a continuous signal not resolved into foci) were shown in graphs. Three gonads were assessed per genotype.

Quantification of TOP-3/MSH-5/RMH-1/OLLAS foci

Foci were quantified by dividing the gonad into four equal zones from the transition zone to late pachynema, and counting the number of foci per nucleus for each zone. For OLLAS::COSA-1, only the number of foci per nucleus in the last zone (zone 4) was determined. At least three gonads were assessed per genotype.

Quantification of early and late TOP-3::HA foci appearance in different genotypes

Five to six *C. elegans* gonads per genotype were divided into cell rows from the beginning of the transition zone (the region where the nuclei display the characteristic half-moon shape) until late pachynema. HA foci appearance was defined as the region where more than 50% of the nuclei displayed discernible foci.

Quantification of meiotic region positive for SUN-1pS8

The gonads used for quantification were divided into cell rows and the region positive for SUN-1pS8 was defined as the region where more than 50% of the nuclei had the signal. The region positive for SUN-1pS8 was normalized to the gonad length from meiotic onset until late pachynema. Five gonads were quantified per genotype.

Viability analysis

Single L4 hermaphrodite were transferred onto individual NGM plates and allowed to lay eggs at 20°C. Worms were shifted every 24 hours onto fresh plates for 3 days. After the worm was removed, all of

the eggs were scored: dead eggs and viable larvae were counted after 24 hours and numbers of males and larval arrests were scored after 72 hours. The hatched rate/embryonic viability was calculated as the number of hatched eggs divided by the total number of laid eggs in percentage, and the rates of males and larval arrest were calculated as the total number of males or larval arrests over the number of hatched eggs in percentage.

Yeast two hybrid assays

Yeast two hybrid assays were conducted according to a published protocol (3). cDNAs encoding TOP-3 and RMH-2 were cloned into the following vectors (via the SacI and XmaI sites for RMH-2 and the SacII and XmaJI sites for TOP-3): two prey vectors pDP133 (containing an N-terminal HA tag) and pDP174 (containing a C-terminal HA tag) and two bait vectors pDP134 (containing a C-terminal LexA tag) and pDP135 (containing a N-terminal LexA tag). The yeast strain MATa (L40) was co-transfected with the pray and bait vectors (both N- terminal and C-terminal) and selected on SC-Leu-Trp plates. Protein interactions were assayed on SC-Leu-Trp-His plates after 2–4 days of culture at 30°C. Growth was assayed on SC-Leu-Trp plates after 2–4 days of culture at 30°C. The experiment was conducted twice and confirmed with two different clones for each combination or bait and prey.

Strain list

Experimental models: organisms and strains		
C. elegans: N2 Bristol	CGC	https://cgc.umn.edu/strain/search
C. elegans: top-3-ZnF(jf153) III	This paper	UV239
C. elegans: top-3::ha(jf158) III	This paper	UV240
C. elegans: top-3-ZnF::ha(jf159) III	This paper	UV241
C. elegans: rtel-1(1866) I	National Bioresource Project, Tokyo, Japan	
C. elegans: rtel-1(tm1866) I; top-3-ZnF::ha(jf159)III	This paper	UV242
C. elegans: mus-81(tm1937) I	(4)	TG1760
C. elegans: mus-81(tm1937) I; top-3-Znf(jf153) III	This paper	UV243
C. elegans: spo-11(ok79)/nT1 (IV;V)	(5)	AV106
C. elegans: spo-11(ok79)/ nT1 (IV;V); top-3-ZnF(jf153) III	This paper	UV244
C. elegans: top-3-ZnF(jf153) III Hw	This paper	UV245
C. elegans: rmh-2(jf168) V	This paper	UV246
C. elegans: rmh-2(jf168) V Hw	This paper	UV247
C. elegans: Hw strain	cgc	CB4856
C. elegans: rtel-1(tm1866) I; top-3::ha(jf158) III	This paper	UV248
C. elegans: top-3::ollas(jf110) III	(6)	UV198
C. elegans: gfp::rmh-1(jfsi38) I	(7)	UV208
C. elegans: him-6::ha(jf93) IV	(7)	UV119
C. elegans: top-3::ollas(jf110) III; rmh-1::gfp(jfsi38) I; him-6::ha(jf93) IV	This paper	UV249
C. elegans: top-3::ollas(jf110) III; rmh-1(jf54)I	This paper	UV250
C. elegans: top-3::ollas(jf110) III; him-6(ok412) IV	This paper	UV251
C. elegans: gfp::rmh-1(jfsi38)/ht2 I; top-3(jf101)/ht2 III; him-6::ha(jf93) IV	This paper	UV252
C. elegans: top-3(jf101)	(8)	UV149
C. elegans: gfp::rmh-1(jfsi38)/ht2 I; top-3-ZnF(jf153) III; him-6::ha(jf93) IV;	This paper	UV253

C. elegans: gfp::rmh-1 (jfsi38) I; top-3-ZnF(jf153) III; rmh-2(jf94) V/ nT1 (IV;V)	This paper	UV254
C. elegans: gfp::msh-5; ollas::cosa-1	(9)	
C. elegans: gfp::msh-5 IV; ollas::cosa-1 top-3-ZnF(jf153)III	This paper	UV255
C. elegans: ollas::cosa-1 III; gfp::msh-5/ nT1 IV; rmh-2(jf94)/ nT1 V	This paper	UV256
C. elegans: ollas::cosa-1 top-3-ZnF(jf153) III; gfp::msh-5/ nT1 IV; rmh-2(jf94)/ nT1 V	This paper	UV257
C. elegans: top-3-ZnF::ha(jf159) III; rmh-2(jf94)/nT1 (IV;V)	This paper	UV258
C. elegans: top-3::ha(jf158) III; rmh-2(jf94)/nT1 (IV;V)	This paper	UV259
C. elegans: rmh-2(jf94)/nT1 (IV;V)	(7)	UV174
C. elegans: top-3-ZnF::ha(jf159) III; spo-11(ok79)/ nT1 IV; rmh-2(jf94)/nT1 V	This paper	UV260
C. elegans: rmh-2::flag(jf197) V	This paper	UV261
C. elegans: rmh-2::flag(jf197) V; top-3-ZnF::ha(jf159) III	This paper	UV262
C. elegans: rmh-2::mCherry	This paper	UV271
C. elegans: rmh-2::mCherry V; top-3-ZnF::ha(jf159) III	This paper	UV272
C. elegans: rmh-2::mCherry V; top-3::ha(jf158) III	This paper	UV273
C. elegans: rmh-1(jf54)	(7)	UV173
C. elegans: rmh-1(jf54) I; top-3-ZnF::ha(jf159) III	This paper	UV263
C. elegans: him-6 (ok412) IV	(10)	VC193
C. elegans: mIn1[mIs14 (GFP)rol-1(e91)]/dpy-25(e817) II	(11)	DW579
C. elegans: top-3-ZnF(jf153) III; mIn1[mIs14 (GFP)rol-1(e91)]/dpy-25(e817) II	This paper	UV265
C. elegans: rmh-2(jf94) V; mIn1[mIs14 (GFP)rol-1(e91)]/dpy-25(e817) II	This paper	UV266
C. elegans: rmh-2(jf168) V; mIn1[mIs14 (GFP)rol-1(e91)]/dpy-25(e817) II	This paper	UV267
C. elegans: top-3-ZnF(jf153) III; rmh-2(jf168) V; mIn1[mIs14 (GFP)rol-1(e91)]/dpy-25(e817) II	This paper	UV268
C. elegans: top-3-ZnF^{4CtoA}(jf219) III	This paper	UV275

1. Madeira, F., Park, Y.M., Lee, J., Buso, N., Gur, T., Madhusoodanan, N., Basutkar, P., Tivey, A.R.N., Potter, S.C., Finn, R.D. *et al.* (2019) The EMBL-EBI search and sequence analysis tools APIs in 2019. *Nucleic Acids Res*, **47**, W636-W641.
2. Link, J., Paouneskou, D., Velkova, M., Daryabeigi, A., Laos, T., Labella, S., Barroso, C., Pacheco Pinol, S., Montoya, A., Kramer, H. *et al.* (2018) Transient and Partial Nuclear Lamina Disruption Promotes Chromosome Movement in Early Meiotic Prophase. *Dev Cell*, **45**, 212-225 e217.
3. Kraft, C., Kijanska, M., Kalie, E., Siergiejuk, E., Lee, S.S., Semplicio, G., Stoffel, I., Brezovich, A., Verma, M., Hansmann, I. *et al.* (2012) Binding of the Atg1/ULK1 kinase to the ubiquitin-like protein Atg8 regulates autophagy. *EMBO J*, **31**, 3691-3703.
4. Kruisselbrink, E., Guryev, V., Brouwer, K., Pontier, D.B., Cuppen, E. and Tijsterman, M. (2008) Mutagenic capacity of endogenous G4 DNA underlies genome instability in FANCJ-defective C. elegans. *Curr Biol*, **18**, 900-905.
5. Dernburg, A.F., McDonald, K., Moulder, G., Barstead, R., Dresser, M. and Villeneuve, A.M. (1998) Meiotic recombination in C. elegans initiates by a conserved mechanism and is dispensable for homologous chromosome synapsis. *Cell*, **94**, 387-398.
6. Velkova, M., Silva, N., Dello Stritto, M.R., Schleiffer, A., Barraud, P., Hartl, M. and Jantsch, V. (2021) Caenorhabditis elegans RMI2 functional homolog-2 (RMIF-2) and RMI1 (RMH-1) have both overlapping and distinct meiotic functions within the BTR complex. *PLoS Genet*, **17**, e1009663.
7. Jagut, M., Hamminger, P., Woglar, A., Millonigg, S., Paulin, L., Mikl, M., Dello Stritto, M.R., Tang, L., Habacher, C., Tam, A. *et al.* (2016) Separable Roles for a Caenorhabditis elegans RMI1 Homolog in Promoting and Antagonizing Meiotic Crossovers Ensure Faithful Chromosome Inheritance. *PLoS Biol*, **14**, e1002412.
8. Dello Stritto, M.R., Bauer, B., Barraud, P. and Jantsch, V. (2021) DNA topoisomerase 3 is required for efficient germ cell quality control. *J Cell Biol*, **220**.
9. Janisiw, E., Dello Stritto, M.R., Jantsch, V. and Silva, N. (2018) BRCA1-BARD1 associate with the synaptonemal complex and pro-crossover factors and influence RAD-51 dynamics during Caenorhabditis elegans meiosis. *PLoS Genet*, **14**, e1007653.
10. Wicky, C., Alpi, A., Passannante, M., Rose, A., Gartner, A. and Muller, F. (2004) Multiple genetic pathways involving the Caenorhabditis elegans Bloom's syndrome genes him-6, rad-51, and top-3 are needed to maintain genome stability in the germ line. *Mol Cell Biol*, **24**, 5016-5027.
11. Leon-Ortiz, A.M., Panier, S., Sarek, G., Vannier, J.B., Patel, H., Campbell, P.J. and Boulton, S.J. (2018) A Distinct Class of Genome Rearrangements Driven by Heterologous Recombination. *Mol Cell*, **69**, 292-305 e296.
